# A single cell transcriptome atlas of the developing zebrafish hindbrain

**DOI:** 10.1242/dev.184143

**Published:** 2020-03-16

**Authors:** Monica Tambalo, Richard Mitter, David G. Wilkinson

**Affiliations:** 1 Neural Development Laboratory, The Francis Crick Institute, 1 Midland Road, London NW1 1AT, UK; 2 Bioinformatics and Biostatistics, The Francis Crick Institute, 1 Midland Road, London NW1 1AT, UK

**Keywords:** Hindbrain segmentation, Dorsoventral patterning, Neurogenesis, Single cell RNA sequencing, Hindbrain boundary, Fgf signalling

## Abstract

Segmentation of the vertebrate hindbrain leads to the formation of rhombomeres, each with a distinct anteroposterior identity. Specialised boundary cells form at segment borders that act as a source or regulator of neuronal differentiation. In zebrafish, there is spatial patterning of neurogenesis in which non-neurogenic zones form at boundaries and segment centres, in part mediated by Fgf20 signalling. To further understand the control of neurogenesis, we have carried out single cell RNA sequencing of the zebrafish hindbrain at three different stages of patterning. Analyses of the data reveal known and novel markers of distinct hindbrain segments, of cell types along the dorsoventral axis, and of the transition of progenitors to neuronal differentiation. We find major shifts in the transcriptome of progenitors and of differentiating cells between the different stages analysed. Supervised clustering with markers of boundary cells and segment centres, together with RNA-seq analysis of Fgf-regulated genes, has revealed new candidate regulators of cell differentiation in the hindbrain. These data provide a valuable resource for functional investigations of the patterning of neurogenesis and the transition of progenitors to neuronal differentiation.

## Abstract (precis)

**Summary:** A single cell transcriptome at three stages of zebrafish hindbrain development provides a valuable resource for investigations of the patterning of neurogenesis and transition of progenitors to neuronal differentiation.

## INTRODUCTION

Development of the central nervous system (CNS) requires precise regulation of the differentiation of neuronal and glial cell types from neural progenitor cells. This is achieved through a network of cell-cell signalling and transcription factors that inhibit or promote cell differentiation and specify cell type along the dorsoventral (D-V) and anteroposterior (A-P) axes of the neuroepithelium. Cell specification along the D-V axis involves localised sources of Shh, BMP and Wnt signals that act in a concentration-dependent manner to regulate expression of specific transcription factors (Dessaud et al., 2008, 2007; Hikasa and Sokol, 2013; Ikeya et al., 1997; Lee and Jessell, 1999; Liem et al., 1997; Panhuysen et al., 2004; Timmer et al., 2002; Ulloa and Martí, 2010). This positional information is integrated with patterning along the anteroposterior axis, which regulates expression of transcription factors that specify regional identity within the brain and spinal cord (Alexander et al., 2009). Differentiation is also under temporal regulation, with distinct neuronal or glial cell types arising at different times (Guillemot, 2007). It is essential that a pool of progenitor cells is maintained as a source of later-differentiating cells, and this is achieved by multiple mechanisms that inhibit differentiation.

The switch of progenitor cells to neuronal differentiation involves the sustained high-level expression of proneural transcription factors that initiate a cascade of gene expression leading to expression of terminal neuronal markers (Bertrand et al., 2002). The expression and function of proneural genes is antagonised by intrinsic factors, as well as by extrinsic signals such as Notch ligands and Fgfs that inhibit differentiation (Fisher and Caudy, 1998; Gonzalez-Quevedo et al., 2010; Kageyama et al., 2005; Ortega et al., 1998; Vaccarino et al., 1999; Zheng et al., 2004). In some regions of the developing CNS, neurogenesis occurs widely in the neuroepithelium, and lateral inhibition due to expression of Notch ligands by differentiating neurons ensures that progenitor cells are maintained (Pierfelice et al., 2011). In other regions, there is a patterning of neurogenesis, e.g. due to spatially restricted expression along the anteroposterior or D-V axis of Hes/Her genes that inhibit neuronal differentiation (Bae et al., 2005; Geling et al., 2003). Studies of the vertebrate hindbrain have revealed further mechanisms that regulate the patterning of neuronal differentiation.

The hindbrain is an important component of the CNS, which includes neurons that innervate cranial muscles, that relay sensory inputs, and that control breathing, the heart and gastrointestinal systems. At early stages, the neuroepithelium of the hindbrain is subdivided to form seven rhombomeres (r1-r7), each expressing a distinct set of transcription factors, including *egr2* (*krox20*), *mafb*, Hnf1 and Hox genes, that underlie segmentation and anteroposterior identity (Alexander et al., 2009). A similar but different set of neurons is generated in each rhombomere (Clarke and Lumsden, 1993; Lumsden, 2004; Lumsden and Keynes, 1989); e.g. in mouse, the Vth, VIIth and IXth branchiomotor nerves form in r2+r3, r4+r5 and r5+r6, respectively. There is a partial understanding of mechanisms that link A-P identity to neuronal cell type specification in the hindbrain (Narita and Rijli, 2009).

Boundary formation has a crucial role in the organisation of neurons and neurogenesis in the hindbrain. Through a combination of cell identity regulation (Addison et al., 2018; Wang et al., 2017) and Eph-ephrin-mediated cell segregation (Batlle and Wilkinson, 2012; Cayuso et al., 2015; Fagotto, 2014), each rhombomere is demarcated by sharp borders and has a homogeneous segmental identity. Specialised boundary cells form at each rhombomere border (Guthrie and Lumsden, 1991), which express specific molecular markers (Cheng et al., 2004; Cooke et al., 2005; Heyman et al., 1995; Letelier et al., 2018; Xu et al., 1995). These boundary cells are induced by Eph receptor signalling that leads to an increase in mechanical tension and activation of Taz (Cayuso et al., 2019). In the chick hindbrain, boundary cells have a lower rate of proliferation (Guthrie et al., 1991) and are Sox2-expressing neural stem cells that are a source of neurogenesis (Peretz et al., 2016). A different situation occurs in the zebrafish hindbrain, in which expression of proneural transcription factors is initially widespread, and later becomes confined to zones flanking hindbrain boundary cells (Amoyel et al., 2005; Cheng et al., 2004). Notch activation promoted by *rfng* expression inhibits neurogenesis at early stages in boundary cells (Cheng et al., 2004). In addition, there is increased proliferation and inhibition of neurogenesis in boundary cells by activation of the Yap/Taz pathway downstream of mechanical tension (Voltes et al., 2019). At late stages (after 40 hpf), proliferation declines and neurogenesis starts to occur in boundary progenitors (Voltes et al., 2019), similar to the situation in chick (Peretz et al., 2016). Neurogenesis is inhibited at segment centres by Fgf20-expressing neurons that act on the adjacent neuroepithelium (Gonzalez-Quevedo et al., 2010). The clustering of Fgf20-expressing neurons at segment centres is maintained by semaphorin-mediated chemorepulsion from boundary cells (Terriente et al., 2012). In addition to suppressing neuronal differentiation, Fgf signalling may switch progenitors at the segment centre to glial differentiation (Esain et al., 2010). The zebrafish hindbrain thus has a precise organisation of signalling sources that underlies a stereotyped pattern of neurogenic and non-neurogenic zones, and the positioning of neurons within each segment.

We set out to identify further potential regulators of neurogenesis during hindbrain segmentation using single cell RNA sequencing (scRNA-seq) to identify genes specifically expressed in distinct progenitors and differentiating cells, prior to and during the patterning of neurogenesis. Analyses of the transcriptome of single cells revealed known genes and new markers of distinct hindbrain segments, of cell types along the D-V axis, and of the transition of progenitors to neuronal differentiation. We also find temporal changes in gene expression, both in progenitors and differentiating cells, at the different stages analysed. By carrying out supervised clustering, we have identified further genes specifically expressed in hindbrain boundary cells and segment centres. These findings are compared with bulk RNA-seq analyses following loss and gain of Fgf signalling to identify potential regulators expressed in segment centres.

## RESULTS

### Single cell profiling of the developing zebrafish hindbrain and surrounding tissues

**Fig. 1.**
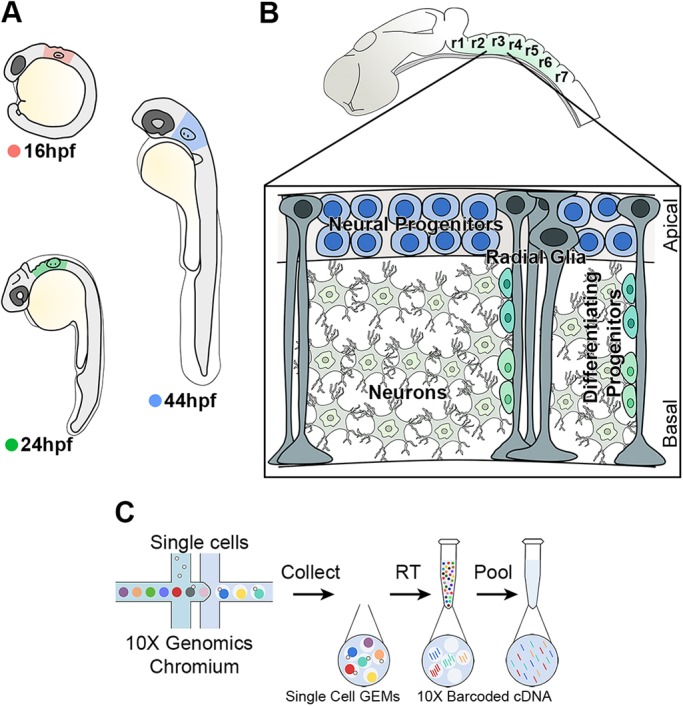
**High-throughput scRNA-seq strategy from the developing hindbrain.** (A) The hindbrain of 16 hpf (pink), 24 hpf (green) and 44 hpf (blue) embryos was collected for scRNA-seq. (B) Drawing of zebrafish hindbrain with a closer view of the stereotypical hindbrain cell composition at 44 hpf. Progenitors and radial glia cell bodies occupy the ventricular region, while differentiating progenitors and neurons are in the mantle zone. (C) Schematic of the 10X Genomics Chromium workflow.

To further understand the progressive patterning of neurogenesis of the developing zebrafish hindbrain, we analysed the transcriptome of single cells at three developmental stages (Fig. 1A,B): 16 hpf (prior to patterning of neurogenesis), 24 hpf (beginning of neurogenic patterning) and 44 hpf (pattern of neurogenic and non-neurogenic zones fully established). For each stage, we micro-dissected the hindbrain territory from around 40 embryos, which were pooled. After enzymatic digestion and mechanical dissociation, the single cell suspension was loaded into the droplet-based scRNA-seq platform 10X Genomics Chromium (Fig. 1C). In total, 9026 cells were sequenced (2929 at 16 hpf, 2568 at 24 hpf and 3529 at 44 hpf), with an average number of UMIs of 6916 and 1703 median genes per cell (Fig. S1).

Seurat unsupervised clustering was used to classify cell population identity (Butler et al., 2018; Stuart et al., 2019) after aggregating the data from all stages (Fig. S2). Cluster projection onto UMAP plots (Becht et al., 2018; McInnes et al., 2018) revealed a tight group of cells with some substructure, and a number of peripheral clusters (Fig. S2A). As the dissections included tissues adjacent to the hindbrain, it is likely that the clusters correspond to distinct tissue types. We therefore used tissue marker genes to assign cluster identity. The progenitor marker Sox3 and neuronal gene *elavl3* were found to mark complementary parts of the main group of cells and together define the hindbrain territory (Fig. S2B,C). This group of cells has a substructure due to changes in transcriptome within and between different stages that will be analysed below. Sox3 also marks a peripheral cluster of hindbrain cells that co-express *shh* (Fig. S2D) and therefore derive from the floor plate. The expression of marker genes reveals that other clusters correspond to tissues found next to the hindbrain, as follows: neural crest (*foxd3* and *twist1a*), head mesenchyme and mesendoderm (*colec12* and *col9a2*), vasculature (*sox7*), pharyngeal arches (*foxi1*), epidermis (*krt17*), otic vesicle (*eya2*), and otic and cranial ganglia (*neurod1*) (Fig. S2A,D). Based on this analysis, we bioinformatically recovered hindbrain cells for each stage: 1678 cells at 16 hpf, 1722 cells at 24 hpf and 2729 cells at 44 hpf (Table S1).

### Overall changes in hindbrain tissue composition

**Fig. 2.**
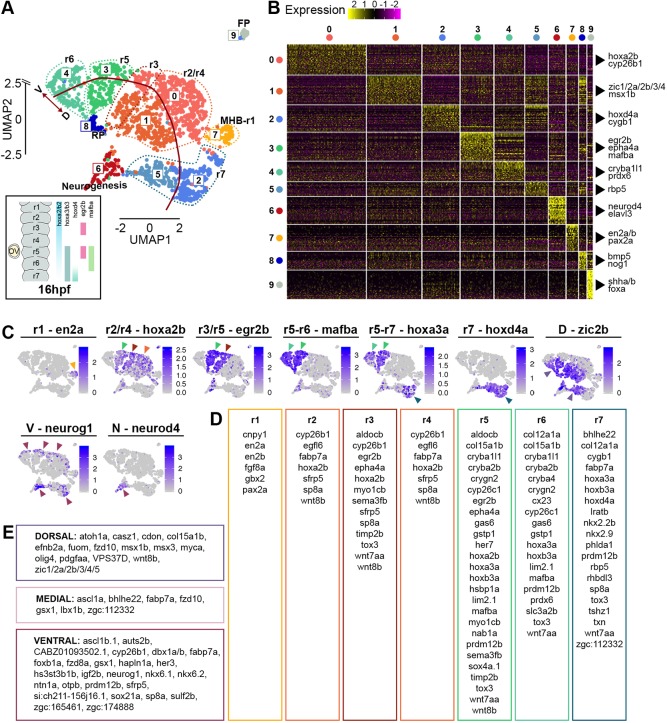
**Cell population composition and signatures of the 16 hpf hindbrain.** (A) An unsupervised UMAP plot subdivides hindbrain cells into 10 clusters (C0-C9). Dotted lines segregate different rhombomeres (r), midbrain-hindbrain boundary (MHB), floor plate (FP), roof plate (RP) and cells undergoing neurogenesis are also highlighted. The red line separates dorsal versus ventral cells. UMAP2 (*y*-axis) is discontinuous. Below the UMAP, a schematic view of the zebrafish hindbrain at 16 hpf and selected segmental genes. (B) Heatmap of the top 30 genes significantly enriched in each cluster; representative gene names are shown close to each cluster. The full gene list is in Fig. S3. (C) UMAP plots showing the log normalised counts of representative genes. Colour intensity is proportional to the expression level. Arrowheads indicate the relevant domain of expression; colour refers to cluster of origin. (D) Summary of rhombomere-specific genes extracted from the top 30 significantly enriched. (E) Summary of genes restricted along the D-V axis.

**Fig. 3.**
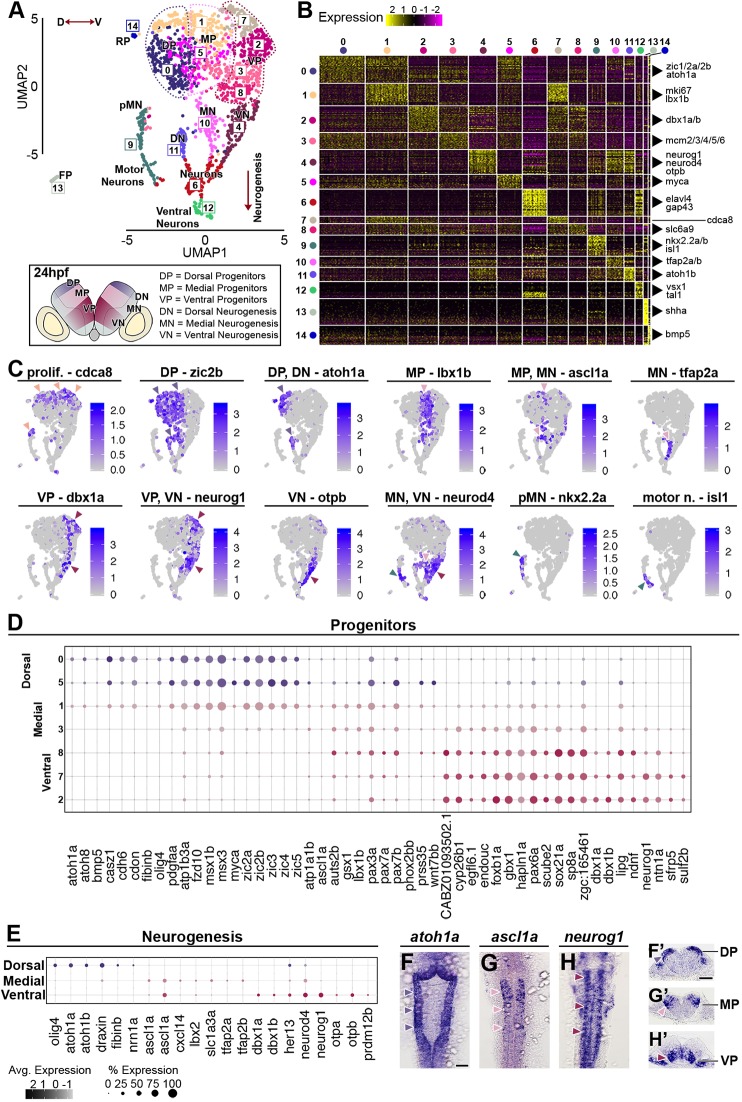
**Cell population composition and signatures of the 24 hpf hindbrain.** (A) Unsupervised UMAP plot subdivides hindbrain cells into 15 clusters. Dotted lines segregate dorsal (dark violet), medial (pink) and ventral (maroon) progenitors; red arrowed lines indicate the D-V axis and the direction of neurogenesis. Below the UMAP, a schematic drawing of a representative transverse section of a 24 hpf zebrafish hindbrain at the level of the otic vesicle (DP, dorsal progenitors; MP, medial progenitors; VP, ventral progenitors; pMN, progenitors motor neurons; DN, dorsal neurogenesis; MN, medial neurogenesis; VN, ventral neurogenesis; FP, floor plate; RP, roof plate). (B) Heatmap of the top 30 genes significantly enriched in each cluster; representative gene names are shown close to each cluster. The full gene list is in Fig. S5. (C) UMAP plots showing the log normalised counts of selective representative genes. Colour intensity is proportional to the expression level of a given gene. Arrowheads indicate the relevant domain of expression; colour refers to cluster of origin. (D) Dot plot of genes with dorsoventral restricted expression in progenitors. (E) Dot plot of factors with restricted expression in differentiating progenitors. Dot size corresponds to the percentage of cells expressing the feature in each cluster, while the colour represents the average expression level. Whole-mount *in situ* hybridisation showing the expression pattern of *atoh1a* (F,F′), *ascl1a* (G,G′) and *neurog1* (H,H′). (F-H) Dorsal view and (F′-H′) 40 µm hindbrain transverse section at the level of r4-r5/r5-r6. Scale bars: 50 µm.

**Fig. 4.**
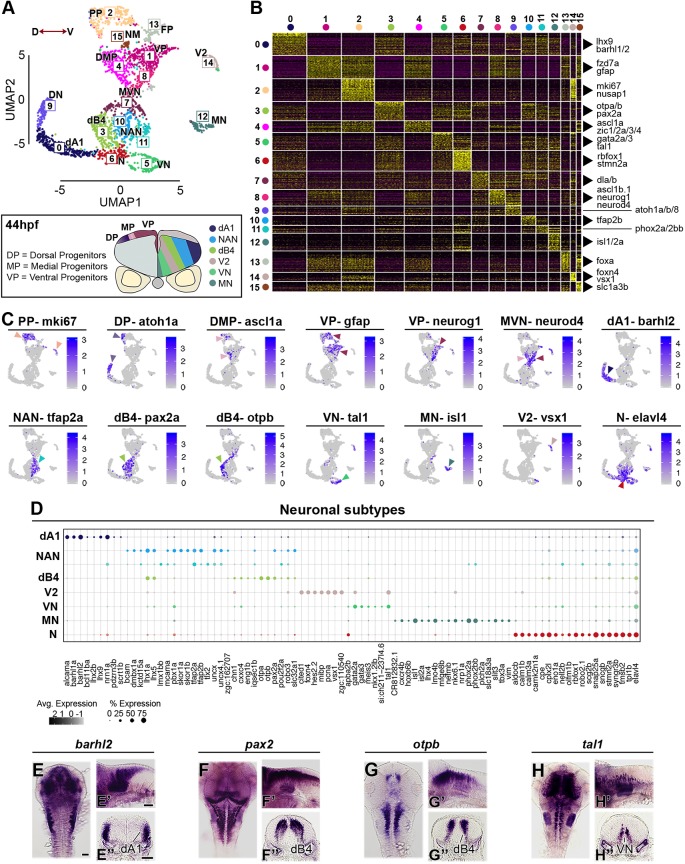
**Neuronal complexity of the 44 hpf hindbrain.** (A) An unsupervised UMAP plot subdivides cells into 16 clusters. Red arrowed line indicates the D-V axis. Below the UMAP is a schematic drawing of a representative transverse section of a 44 hpf zebrafish hindbrain at the level of the otic vesicle (PP, proliferative progenitors; DMP, dorsomedial progenitors; VP, ventral progenitors; MVN, medio-ventral neurogenesis; DN, dorsal neurogenesis; dB4, GABAergic interneurons; NAN, noradrenergic neurons; dA1, dorsal neurons; N, neurons; VN, ventral neurons; V2, interneurons; MN, motor neurons; FP, floor plate; NM, neuromast). (B) Heatmap of the top 30 genes significantly enriched in each cluster, representative gene names are shown close to each cluster. For the full gene list, refer to Fig. S6. (C) UMAP plots showing the log normalised counts of selective representative genes. Colour intensity is proportional to the expression level of a given gene. Arrowheads point to the relevant domain of expression; colour refers to cluster of origin. (D) Dot plot showing neuronal subtype molecular signature. Dot size corresponds to the percentage of cells expressing the feature in each cluster, while the colour represents the average expression level. Whole-mount *in situ* hybridisation showing the expression pattern of *barhl2* (E,E′), *pax2* (F,F′), *otpb* (G,G′) and *tal1* (H-H′). (E-H) Dorsal view, (E′-H′) lateral view and (E″-H″) 40 µm hindbrain transverse section at the level of r4-r5/r5-r6. Scale bars: 50 µm.

We used an unsupervised graph-based clustering approach to analyse the transcriptome data at 16 hpf, 24 hpf and 44 hpf. Datasets were visualised with UMAP dimensionality reduction, and this revealed unique features that reflect the greatest transcriptomic differences between cell types at each developmental stage (Figs 2A, 3A and 4A). Analysis of the top 30 significantly enriched genes per cluster, and expression of known molecular markers, enabled each cluster to be identified. The 16 hpf hindbrain is mainly constituted of progenitors (91% of total hindbrain cells), and cells at different stages of neurogenesis (*neurod4*, elevated *neurog1* expression) account for 6% of hindbrain cells (cluster C6 in Fig. 2A). Progenitors remain the most abundant hindbrain cell type at 24 hpf (71% of hindbrain cells), while 28% of cells express markers of different stages of neurogenesis and late differentiation (C4, C6, C9, C10, C11, C12 in Fig. 3A). By 44 hpf, the proportion of progenitor cells has further diminished to 40%, with 55% of the cells expressing markers of neurogenesis and late stages of neuronal differentiation (C0, C3, C5, C6, C7, C8, C9, C10, C11, C12 and C14 in Fig. 4A). The clustering of cells by transcriptomic differences changes at the three stages. At 16 hpf, clustering is mainly driven by segmental and D-V identity (Fig. 2A), whereas at 24 hpf and 44 hpf cells are clustered by D-V identity and differentiation state (Fig. 3A; Fig. 4A). This change reflects the greater proportion of cells undergoing differentiation at the later stages, with an increasing number of neuronal subtypes by 44 hpf (Fig. 4A). Below, we present more detailed analyses of each of these features that reveal known genes and novel markers of segmental identity, D-V identity and differentiation state. An annotated list, including information on any previous studies of these genes is presented in Table S2.

### Transcriptional signatures of hindbrain segments

The expression of known markers enables the identity of all clusters (C0-C9) at 16 hpf to be deduced (Fig. 2A). At this stage, the main features that drive clustering of hindbrain cells are segmental identity and D-V identity of progenitors, and one cluster of cells (C6) undergoing neurogenesis. We display the genes that distinguish the different clusters in a heatmap of the top 30 differentially expressed genes (Fig. 2B; Fig. S3) and show the expression level of selected genes in UMAP projection plots that relate them to the Seurat analysis (Fig. 2C). Genes specifically expressed in different hindbrain rhombomeres (r), or in dorsal, medial or ventral domains, are listed in Fig. 2D,E, respectively. The single cell gene expression strongly correlates with *in situ* hybridisation data deposited in ZFIN. Information on any previous studies of these genes is presented in Table S2.1.

UMAP projection plots with dorsal and ventral marker genes (Fig. 2C,E) reveal the relationship between D-V identity and the clustering of cells in Seurat. For example, *zic2b* expression marks the dorsal part of all hindbrain segments, and *neurog1* marks ventral progenitors as well as differentiating neurons (Fig. 2A,C). For some hindbrain segments (r2, r3, r4 and r7) but not others (r1, r5 and r6), cells with distinct D-V identity segregate into discrete clusters; this presumably reflects the quantitative difference in transcriptome in relation to the threshold for assigning cells to different clusters. Indeed, increasing cluster resolution further subdivides the hindbrain territory in a total of 19 clusters (Fig. S4A), with increased segregation into dorsal, medial and ventral populations (Fig. S4B). Roof plate cells (C8) in the dorsal-most neuroepithelium form a discrete cluster, expressing markers including *bmp5* and *nog1* (Fig. 2B), that is adjacent to cells expressing dorsal markers (Fig. 2A). As also seen in the aggregated data (Fig. S2), floor plate cells form a cluster (C9) that is distant in UMAP from other hindbrain cells.

The clustering of cells based on segmental identity is revealed in projection plots of selected marker genes that are expressed in different sets of segments (Fig. 2C): *en2a* (MHB-r1), *hoxa2* (r2-r5), *egr2b* (r3, r5), *mafba* (r5, r6), *hoxa3* (r5-r7) and *hoxd4a* (r7). Cells from r2, r3 and r4 co-cluster in C0-C1, where C0 cells are ventral and C1 cells are dorsal (Fig. 2A). Seurat analysis did not discriminate r2 and r4 cells, suggesting strong transcriptional similarities, including *egfl6*, *fabp7a* and *sfrp5* expression (Fig. 2D). Cells from r3 are included in C0-C1, but form a discrete group that is marked, e.g. by *egr2b* expression (Fig. 2A,C,D). This clustering of r2, r3 and r4 cells reflects that genes including *hoxa2b*, *sfrp5* and *sp8a* are expressed in all three segments, whereas *egr2b*, *epha4a*, *sema3fb* and other markers are expressed in r3 cells (Fig. 2C,D). After increasing cluster resolution, r3 becomes segregated from r2 and r4 (Fig. S4A). Consistent with previous studies, r3 cells are adjacent to r5 cells (C3), reflecting that they express some genes in common: in addition to the extensively-studied *egr2b* and *epha4a* genes, they express *timp2a*, *aldocb*, *sema3fb* and *myo1cb* (Fig. 2D). r5 also shares transcriptional similarities with r6, which forms an adjacent cluster (C4), including *mafba* (Fig. 2C), *cryba2b*, *crygn2*, *lim2.1*, *col15a1b* and *gas6*. However, r7 cells (C2 ventral and C5 dorsal) do not cluster adjacent to r6 cells, reflecting that, although some genes are expressed in both segments (e.g. *hoxa3a*, *hoxb3a* and *tox3*), many other genes are expressed in one or the other, e.g. *hoxd4a* (Fig. 2C), *fabp7a*, *lratb*, *rbp5*, *rhbdl3* and *sp8a* in r7 (Fig. 2D). r1 and midbrain-hindbrain boundary (MHB) cells, which express known markers [*eng2a/b* (Fig. 2C), *fgf8a, cnpy1* and *pax2a*], are found to cluster together in C7. As summarised in Table S2.1, these analyses have identified genes not previously described to have segmental expression in the hindbrain; these include *myo1cb* and *timp2b* in r3 and r5. In addition, we found genes for which expression data are available, but have not been tested functionally in the hindbrain; these include *sp8a* (strong in r4 and r7, weak in r2 and r3), *sfrp5* (r2-r4) and *wnt7aa* (r3-r7).

We further analysed the transcriptome data using PlotClusterTree in Seurat as this better represents the similarity between clusters than UMAP distance. Analysis of the 16 hpf data at higher cluster resolution (Fig. S4C) segregates cells with distinct segmental identity, D-V identity and differentiation state. Cells from r2, r3 and r4 are found to be closely related and are further subdivided based on D-V rather than segmental identity: ventral r3 and ventral r2+r4 form adjacent branches, and dorsal r3 and dorsal r2+r4 form adjacent branches. This suggests that dorsoventral identity underlies greater transcriptomic similarities between cell clusters than segmental identity within this population. The tree analysis reveals further clusters of r2+r4 cells (3 and 6) that in heat maps are found to have higher expression of genes related to cell proliferation. The tree analysis suggests that r5, r6 and most r7 cells are closely related and subdivides them into sequential and discrete branches, each further subdivided into dorsal and ventral populations. Some cells classified as r7 (cluster 15) form a separate branch; however, we find that these do not express *hoxd4a*, and may correspond to spinal cord cells caudal to the hindbrain. Finally, the MHB-r1, roof plate, differentiating neurons and floor plate form separate branches.

### Dorsoventral signatures of progenitors and differentiating neurons

D-V positional information regulated by BMP, Wnt and Shh signalling is a key feature of the developing neuroepithelium that underlies specification of neuronal cell types. Extensive molecular characterisation has been carried out in the spinal cord (Delile et al., 2019; Gouti et al., 2015), but less widely for the hindbrain. At all stages analysed, progenitors were clustered based on their D-V identity, reflecting that D-V patterning is established early and maintained during hindbrain neurogenesis. Seurat analysis at 16 hpf segregates cells into dorsal and ventral progenitors, as well as roof plate and floor plate (Fig. 2A). However, UMAP projection plots with known markers (listed in Fig. 2E) and increasing cluster resolution (Fig. S4) reveal that these are further subdivided into dorsal, medial and ventral domains. Seurat analysis at 24 hpf and 44 hpf clusters cells into dorsal, medial and ventral populations, plus roof plate and floor plate (Figs 3A and 4A). In addition, progenitor cells are further segregated based on expression of proliferation markers. Selected genes that mark these different populations are presented in UMAP projection plots (Figs 3C and 4C), in dot plots of relative expression levels in progenitors and differentiating neurons (Fig. 3D,E; Fig. 4D), and *in situ* hybridisation analyses (Fig. 3F-H; Fig. 4E-H). We describe the 24 hpf and 44 hpf data in more detail below.

At 24 hpf, clusters C0, and part of C1 and C5 are found to express known markers of dorsal progenitors, including *zic2b* (Fig. 3A,C), other Zic genes (Elsen et al., 2008; Grinblat and Sive, 2001), *msx1b/msx3* (Miyake et al., 2012) and *olig3/olig4* (Tiso et al., 2009) (Fig. 3B,D). In addition, these cells express novel markers, including *casz1*, *cdon*, *fzd10*, *myca* and *pdgfaa* (Fig. 2B,D; Fig. S5; Table S2.2). C1 is distinguished from C0 and C5 by expression of proliferation markers, including *cdca8* (Fig. 3A,C). Expression of dorsal markers, including *zic2b*, is also detected in dorsal differentiating neurons (C11, Fig. 3A,C). Dorsal progenitors in C0 and C1, and dorsal differentiating neurons (C11) express the proneural gene *atoh1a* (Fig. 3C,E) (Elsen et al., 2009), which we verified by *in situ* hybridisation (Fig. 3F,F′). Medial progenitors are found in a subset of cells in C1, C3 and C5, sharing a few dorsally (e.g. Zic genes) and ventrally expressed (e.g. *foxb1a* and *pax6a*) factors, while uniquely expressing markers including *gsx1*, *pax7a/pax7b*, *ptf1a* and *lbx1b* (Fig. 3C,D; Fig. S5; Table S2.2). This analysis further shows that the proneural gene *ascl1a* is expressed medially in hindbrain progenitors (C1, C5) and differentiating neurons (C10), with expression overlapping with *neurod4* (Fig. 3C,E; *in situ* hybridisation in Fig. 3G,G′). Ventral progenitors are subdivided into multiple clusters (C7, C2, C3 and C8). Cells in C7 and C3 express higher levels of factors involved in the cell cycle, e.g. Mcm genes (*mcm2-mcm6*), while ventrally restricted genes are enriched in C2 (e.g. *dbx1a/dbx1b*) and C8 (e.g. *irx3a*) (Fig. S5). Overall, we found a ventral progenitor signature in which they express a unique set of transcription factors: *sox21a*, *foxb1a*, *sp8a* and *dbx1a/dbx1b*. These ventral progenitors and differentiating neurons express the proneural gene *neurog1* (Fig. 3C,E), which we verified by *in situ* hybridisation (Fig. 3H,H′). In addition, these cells express several signalling modulators: *sfrp5* (soluble inhibitor of Wnt signalling), *cyp26b1* (RA degradation), *scube2* (Shh long-range signalling) and *sulf2b* (heparan sulfate proteoglycans) (Fig. 3D), which may contribute to modulation of Wnt, RA and Shh levels that underlie neuronal cell type specification (Dessaud et al., 2008; Lara-Ramírez et al., 2013; Lupo et al., 2006; Ulloa and Martí, 2010). Analysis at 44 hpf also clusters progenitor cells based on D-V identity marked by Zic genes, *ptf1a*, *lbx1b*, *dbx1a*, and the proneural genes *ascl1* and *neurog1* (Fig. 4A-C; Fig. S6; Fig. S7). The major feature that has emerged by this stage is differentiation to form a number of neuronal cell types that are described below.

### Characterisation of neuronal complexity

**Fig. 5.**
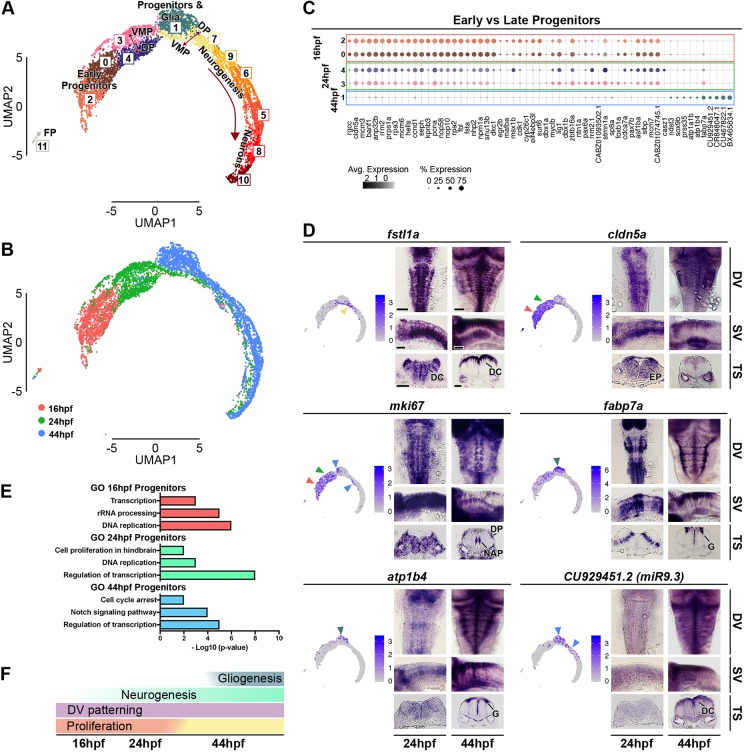
**Analysis of aggregated 16 hpf, 24 hpf and 44 hpf data.** (A) Unsupervised UMAP plot of cells from 16 hpf, 24 hpf and 44 hpf subdivides them into 12 clusters (DP, dorsal progenitors; VMP, ventral-medial progenitors; FP, floor plate). (B) UMAP plots with cells coloured based on their stage of origin: 16 hpf (pink), 24 hpf (green) and 44 hpf (blue). (C) Dot plot showing molecular signature of dorsal and ventral progenitors at the three stages. Dot size corresponds to the percentage of cells expressing the feature in each cluster, while the colour represents the average expression level. The full gene list of top 30 significantly enriched factors is in Fig. S8. (D) UMAP plots showing the log normalised counts of representative genes. Colour intensity is proportional to the expression level of a given gene. Whole-mount *in situ* hybridisation showing the expression pattern of *cldn5a*, *fstl1a*, *mki67*, *fabp7a*, *atp1b4* and *CU929451.2* (*miR9.3*) at 24 hpf and 44 hpf. Arrowheads indicate relevant domains of expression; colour refers to the cluster of origin. Dorsal view (DV), side view (SV) and 40 µm hindbrain transverse section (TS) at the level of r4-r5/r5-r6 are shown for each gene. Scale bars: 50 µm. EN, early neurogenesis; EP, early progenitors; DC, differentiating cells; DP, dorsal progenitors; NAP, non-apical proliferation; G, glia. (E) Selected Gene Ontology (GO) terms at 16 hpf (pink), 24 hpf (green) and 44 hpf (blue) are shown. *x*-axis is -log10 (*P*-value). (F) Summary of global hindbrain changes along the temporal axis.

Different neuronal subtypes are progressively generated from the distinct D-V progenitor domains. At 16 hpf, Seurat analysis identifies a single cluster (C6) expressing markers of neurogenesis (Fig. 2A), and at 24 hpf and 44 hpf identifies distinct clusters that express early and late markers of neuronal differentiation (Figs 3A and 4A). To determine whether the transcriptome of differentiating cells is similar or different at 16, 24 and 44 hpf, we aggregated the data from all stages and carried out Seurat analysis. Unsupervised clustering identifies 12 clusters and separates progenitors (C0, C2, C3 and C4), progenitors and glia (C1), neurons at different stages of differentiation (C5, C6, C7, C8, C9 and C10), and the floor plate (C11) (Fig. 5A; Fig. S8). When cells are labelled by their developmental stage (Fig. 5B), we found that some cells at different stages of neuronal differentiation at 16 hpf overlap with cells at 24 hpf and 44 hpf. Interestingly, they express the activin-binding protein *fstl1a* (Fig. 5D) as well as transcriptional regulators, including *ebf2* (Fig. S8). Likewise, there is some overlap of neurogenesis and neuronal cell types at 24 hpf with differentiating cells at 44 hpf. However, most of the differentiating cells at 24 hpf and 44 hpf are segregated from cells at the earlier stages, consistent with the generation of new neuronal cell types. There are also shifts in the transcriptome of progenitor cells, which will be discussed below.

To characterise the neuronal complexity at 44 hpf (Fig. 4A-D), we classified neuronal subtypes based on previous work (Hernandez-Miranda et al., 2017; Lu et al., 2015). Dorsal progenitors expressing *atoh1a* (C9 in Fig. 4A,C) generate dA1 excitatory interneurons (C0) in the hindbrain, a heterogeneous population that functions in sensory information processing (Hernandez-Miranda et al., 2017). *barhl1a*, *barhl2* (Fig. 4C; *in situ* hybridisation in Fig. 4E; Table S2.3), *lhx2b* and *lhx9* are among their known markers, and in addition we found *alcama*, *bcl11ba* (BAF chromatin remodelling complex), *pdzrn3b* and *scrt1b* (Fig. 4D). Noradrenergic neuron (NAN) development (C10 and C11) is marked by expression of *tfap2a* (Fig. 4C; Table S2.3), which is important for activation of key NA enzymes (Holzschuh et al., 2003; Kim et al., 2001). These cells also express the transcription factors *dmbx1a*, *lhx1a*, *lhx5* and *lbx2*. Interestingly, the two clusters of NAN cells are distinguished by expression of several transcription factors, including *lmx1bb*,* tlx2*,* phox2a* and *phox2bb* (Fig. 4D; Fig. S6; Table S2.3). Another class of neurons found in the hindbrain are GABAergic inhibitory interneurons (dB4), here clustered in C3 (Fig. 4A). These cells express *pax2*, *lhx1* and *lhx5* (Fig. 4D,F; Fig. S6; Table S2.3), which may constitute a transcription factor code (Burrill et al., 1997; Gross et al., 2002; Müller et al., 2002; Pillai et al., 2007). A subset of these cells co-expresses *otpa/otpb* (Fig. 4B-D; *in situ* hybridisation in Fig. 4G; Table S2.3), transcription factors involved in dopaminergic neuron specification (Fernandes et al., 2013), suggesting heterogeneity at this stage. More ventrally (C5), neurons are marked by *tal1* (Fig. 4B-D; *in situ* hybridisation in Fig. 4H) and *gata2a/gata3* expression (Fig. 4D; Table S2.3), resembling ventral neurons identified in the spinal cord (Andrzejczuk et al., 2018). A further cluster of ventral neurons is C14, which expresses *vsx1*, *tal1* and *foxn4* (Fig. 4C,D; *in situ* hybridisation of *tal1* in Fig. 4H; Table S2.3), defining this domain as V2 interneurons. *vsx1*-expressing cells in the hindbrain and spinal cord have been defined as non-apical progenitors, able to generate one excitatory (V2a) and one inhibitory (V2b) interneuron, and have been proposed to be a pool important for rapid generation of the sensory-locomotor circuit (McIntosh et al., 2017); their molecular signature is reported in Fig. 4D. Motor neurons can be identified in C12 (*isl1*, *isl2* and *phox2a*), and in the hindbrain, *lhx4*, *nkx6.1* and *tbx3a* are further transcription factors expressed in these cells (Fig. 4D; Table S2.3). A further neuronal cluster (C6) expresses a specific combination of genes (e.g. *aldocb*, *calm1b*, *camk2n1a* and *rbfox1*; Fig. 4C,D; Table S2.3), but could not be classified. C15 consists of lateral line neuromast cells that were present in the dissected tissue and had not been removed bioinformatically. Our transcriptome atlas thus gives new insights into factors expressed in different neuronal cell types in the hindbrain.

### Transcriptional shift of hindbrain progenitors

In addition to finding temporal differences in expression of neurogenic markers, Seurat analysis of the aggregated data found that 16, 24 and 44 hpf progenitors are in largely distinct clusters in UMAP space (Fig. 5A,B). Analysis of the top 30 significant enriched genes per cluster highlights transcriptional similarities and differences between progenitors (Fig. S8). Genes enriched in both dorsal and ventral progenitors at 16 hpf (C0 and C2) and 24 hpf (C3 and C4) include *cldn5a*, *fsta* and proliferative markers such as *pcna* (Fig. 5C; Fig. S8; Table S2.4). Gene ontology terms associated with the top 30 genes enriched in these progenitors highlight their proliferative property (Fig. 5E). A drastic reduction in proliferation has taken place by 44 hpf. As examples, we show that *mki67*, *nusap1*, *ccnd1* and *cdca8* are widely expressed in the early hindbrain, whereas they are restricted to a small proportion of dorsal progenitors and *vsx1*-expressing cells at 44 hpf (Fig. 5D; Fig. S9). In addition, genes associated with cell cycle arrest (*cdkn1ca* and *cdkn1cb*) and the Notch signalling pathway have increased expression at 44 hpf (Fig. 5E). Glial cells become apparent at 44 hpf in the medio-ventral progenitor pool marked by *fabp7a* (C1), and we find they also express *atp1b4* and *atp1a1b* (Fig. 5D; Fig. S8; Table S2.4). Furthermore, *miR9* loci are detected only at 44 hpf (*miR9.1 CR848047.1*, *miR9.3 CU929451.2* and *miR9.6 CU467822.1*) (Fig. 5C,D; Table S2.4), when they are known to play a key role in the timing of neurogenesis (Coolen et al., 2013, 2012). Overall, this analysis highlights that there are significant temporal changes in gene expression in progenitors between 24 hpf and 44 hpf in the developing hindbrain (Fig. 5F).

### Boundary cell and segment centre progenitors

**Fig. 6.**
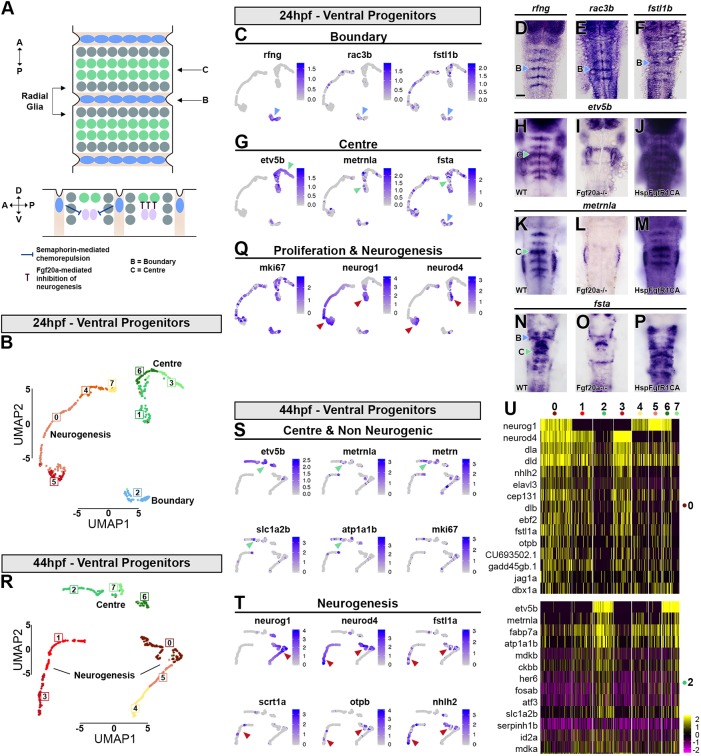
**Transcriptional signature of boundary cells and segment centre progenitors.** (A) Schematic drawing representing anterior-posterior organisation within hindbrain segments. Boundary cells are in blue, neurogenic progenitors in grey and segment centre cells in green. Below is a side view showing the role of boundary cells in maintaining Fgf20a neurons (pink) at the centre of each segment, mediated by semaphorins. Fgf20 signalling maintains undifferentiated progenitors. (B) Supervised clustering of 24 hpf ventral progenitors. Eight clusters are identified: C7, C4 and C0 are progenitors; C5 is the neurogenic domain; C2 are boundary cells; and C1, C6 and C3 are segment centre progenitors. (C,G,Q) UMAP plots showing the expression distribution of boundary (C), segment centre (G) and proliferation and neurogenic genes (Q). Arrowheads indicate relevant domain of expression; colour refers to cluster of origin. (D-F,H,K,N) Whole-mount *in situ* hybridisation of boundary (D-F) and segment centre genes (H,K,N). (I,J,L,M,O,P) Segment centre-specific gene expression is dependent on Fgf20 signalling, as *fgf20a*^−/−^ embryos have loss of *etv5b* (I), *metrnla* (L) and *fsta* (O) expression, whereas constitutive activation of FgfR1 induces their ectopic expression (J,M,P). (R) Supervised clustering of 44 hpf ventral progenitors. Eight clusters are identified: C4 and C5 are progenitors; C0, C1 and C3 are neurogenic domains; C2, C7 and C6 are segment centre progenitors. (S,T) UMAP plots showing the expression distribution of segment centre and non-neurogenic genes (S) and neurogenic genes (T). Arrowheads indicate relevant domain of expression; colour refers to cluster of origin. (U) Heatmap of the top 15 genes enriched in each cluster.

During hindbrain development in zebrafish, proneural gene expression becomes confined to zones flanking the segment boundaries, with low expression in hindbrain boundary cells and also in rhombomere centres (Amoyel et al., 2005; Cheng et al., 2004; Gonzalez-Quevedo et al., 2010). The progenitors at these locations are classified as non-neurogenic as they have low expression of proneural genes required for neuronal differentiation, although this has not been directly shown by lineage analysis (Fig. 6A). The inhibition of neurogenesis has been shown to involve Notch activation (Cheng et al., 2004) and Yap/Taz nuclear translocation (Voltes et al., 2019) at boundaries, and Fgf20 signalling at segment centres (Gonzalez-Quevedo et al., 2010). These distinct progenitor populations were not identified by unsupervised clustering, because this is dominated by the large differences in the transcriptome during D-V patterning and differentiation. We therefore used supervised clustering with known markers to reveal the transcriptional signature of the neurogenic and non-neurogenic cell populations.

We bioinformatically isolated 24 hpf ventral progenitors and used *rfng* (boundary), *etv5b* (segment centre), and *neurog1* and *neurod4* (neuronal differentiation) to drive clustering. Three clusters were obtained that are divided into eight subclusters (Fig. 6B): C2 corresponds to boundary cells (Fig. 6C), C1, C3 and C6 to segment centres (Fig. 6G), and C0, C4, C5 and C7 to neurogenic cells (Fig. 6Q). The neurogenic cells form a continuum in which there is increasing expression of proneural genes and decreased expression of a proliferation marker *mki67* (Fig. 6Q). We found that boundary cells that express *rfng* (C2; Fig. 6D) also express some previously known markers (Fig. S10; Table S2.5): *rasgef1ba* (Letelier et al., 2018) and the Rho GTPase *rac3b* (Fig. 6E; Letelier et al., 2018). In addition, we find new genes with expression enriched at boundaries, including *rnd2, prdm8*, *gsx1* and *grasp* (*tamalin*). We noticed that the BMP inhibitor *follistatin 1b* (Fig. 6F; Dal-Pra et al., 2006) is enriched both in segment centres and boundary cells (Fig. S10), and *in situ* hybridisation analysis confirmed the increased expression at boundaries (Fig. 6F). Thus, we identified a distinct set of factors present in boundary cells with potential functional implications.

At each segment centre, cells respond to Fgf20 signalling and upregulate the Fgf-direct target *etv5b* (Esain et al., 2010; Gonzalez-Quevedo et al., 2010), which we used to drive clustering of 24 hpf progenitors. *etv5b*-expressing cells are found in three adjacent clusters: C1, C3 and C6. In C1 there is transcriptional overlap of *etv5b* with *neurog1*, *ascl1b.1* and *neurod4* (Fig. 6G,Q; Table S2.5), while proneural genes are expressed at a low level in C6 and not detected in C3 cells. The overlapping expression in C1 and C6 likely reflects that, at 24 hpf, *etv5b* is expressed in stripes located at the centre of each segment (Fig. 6H; Table S2.5) but neurogenic gene expression has yet to be fully downregulated (Fig. 3H; Gonzalez-Quevedo et al., 2010). Many of the genes co-expressed with *etv5b* (Fig. S10) have an unknown expression pattern, but based on previous work (Gonzalez-Quevedo et al., 2010), we reasoned that all segment centre marker genes would be under the control of Fgf20. We therefore performed a bulk RNA-seq experiment comparing wild-type dissected hindbrain with *fgf20a* mutant tissue (Gonzalez-Quevedo et al., 2010) (Fig. S11). *metrnla*, which is present in C1 and C6 (Fig. 6G), was among the downregulated genes in *fgf20a*^−/−^ mutants, and *in situ* hybridisation revealed its expression in segment centres (Fig. 6K). The *fgf20a*^−/−^ RNA-seq screen also found *fsta*, which is present in all clusters (Fig. 6G); *in situ* hybridisation suggested this gene has complex expression patterns that include segment centre cells (Fig. 6N). However, *etv5b* was not found in this screen, which likely reflects that it has a complex expression pattern in the hindbrain, otic vesicle and cranial ganglia (Table S3). We therefore also profiled transgenic hindbrains expressing heat-shock-induced constitutively activated FgfR1 [Tg(*hsp70:ca-fgfr1*)] and compared with heat-shocked counterparts (Table S4). This screen found *etv5b*,* metrnla* and *fsta* among the top genes induced by Fgf signalling (Fig. S12). *In situ* hybridisation confirmed that Fgf20 signalling is both necessary and sufficient for expression of *etv5b*, *metrnla* and *fsta* in segment centres (Fig. 6H-P). *metrnla* encodes a cytokine with an unknown receptor. As the related meteorin gene (*metrn*) has been implicated in gliogenesis in other contexts (Lee et al., 2010; Nishino et al., 2004), it is a candidate for promoting glial cell differentiation that occurs at segment centres. Interestingly, *fsta* is also expressed by boundary cells, and thus correlates with non-neurogenic progenitors. Overall, we found a limited number of genes that are exclusively expressed by boundary or centre progenitors, while the majority of transcripts are expressed in the two cell populations (Fig. S10), suggesting similarities in their transcriptome.

**Fig. 7.**
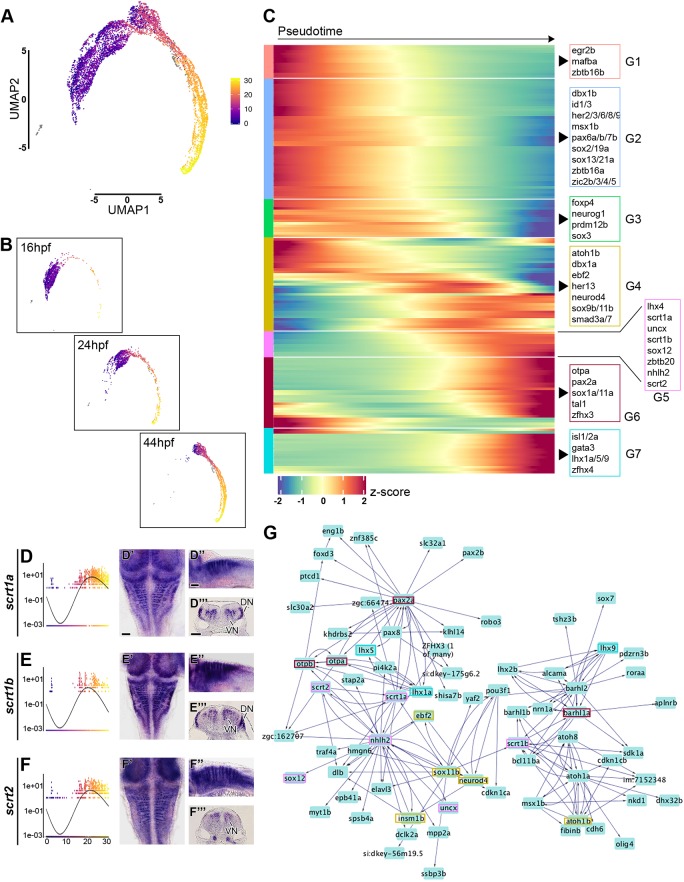
**Analysis of transcription factor expression during hindbrain neurogenesis.** (A) Monocle3 pseudo-temporal ordering of 16 hpf, 24 hpf and 44 hpf hindbrain cells superimposed onto the aggregate UMAP. Cells are coloured based on their progression along pseudotemporal space (from pseudotime 0 in violet to the end of differentiation in yellow). (B) Individual pseudotemporal plots representing cell distribution at each developmental stage. (C) Heatmap showing selected TFs clustered by pseudotemporal expression pattern (q values<0.01). Pseudotime ordering is from left (progenitor state) to right (differentiated neurons). Selected transcription factors are shown for each group (G1-G7). The full gene list is in Fig. S13. (D-F) Expression of *scrt1a*, *scrt1b* and *scrt2* during pseudotime. Whole-mount *in situ* hybridisation at 44 hpf for Scratch genes is shown in dorsal view (D′-F′), side view (D″-F″) and hindbrain sections (D‴-F‴). Scale bars: 50 µm. VN, ventral neurogenesis; DN, dorsal neurogenesis. (G) Using GENIE3, a directed network of interactions was predicted among the genes in the 44 hpf scRNA-seq dataset. The Scratch genes network was viewed and extracted in Cytoscape; boxes highlight TFs present in the above heatmap and colours match the group of origin in C.

At 44 hpf, neurogenic zones are fully refined but *rfng* and other boundary cell markers are no longer detected. We therefore used only *etv5b* and *neurog1+neurod4* to drive clustering. At this stage, *etv5b*-expressing cells segregate together in three adjacent subclusters (C2, C6 and C7) and the overlap with neurogenic genes has greatly decreased (Fig. 6R-U). *metrn* and *metrnla* are expressed in a similar pattern to *slc1a2b*, *atp1a1b* and other glial markers, further suggesting that the Metrn family could play a role in hindbrain gliogenesis. Neurogenic cells segregate into two clusters that are further subdivided: C4, C5 and C0 have a gradient of *neurog1* and *neurod4* expression, suggestive of the progression of neuronal differentiation, while C3 and C1 express only *neurod4*, suggestive of late differentiation (Fig. 6R). These latter cells present a unique signature (Fig. 6U) that includes the expression of *fstl1a* (Fig. 5D; Fig. 6T), and the transcription factors *scrt1a*, *scrt2* (Fig. 6T; Fig. 7C,E) and *nhlh2* (Fig. 6T; Table S2.6).

### Transcription factors temporally regulating hindbrain neurogenesis

To illustrate developmental insights that can be extracted from the single cell RNA-seq data, we focussed on transcription factors (TFs) (AnimalTFDB3.0 database; Zhang et al., 2012) and inferred their potential contribution to hindbrain neurogenesis. We used the aggregated dataset (Fig. 5A) and performed pseudotime analysis using Monocle v3.0.2 (Qiu et al., 2017; Trapnell et al., 2014), which orders cells uniquely on the similarity of their global TF expression profiles. This created a pseudotime trajectory with three discrete cell states (Fig. S14). The root of the trajectory was defined as the state containing the majority of the 16 hpf progenitor cells. The three states are characterised by the expression of: *sox2*, *egr2b*, *mafba*, Zic genes, *pax6a/b* and *zbtb16a/b*, among others, for the progenitor state; *sox3*, *neurog1*, *atoh1a*, *dbx1a*, *gsx1* and *lbx1b* are in the intermediate differentiation state; *atoh1b*, *neurod4*, *isl1*, *vsx1*, *tal1*, *pax2a* and other neuronal transcription factors have high expression level in the final state (Fig. S14). Along the trajectory, cells are ordered largely based on developmental stage of origin and state of differentiation (Fig. 7A,B). 16 hpf and 24 hpf progenitors are found mainly at the start of the trajectory, followed by 44 hpf progenitors. 16 hpf differentiating cells present a TFs expression pattern that mostly resemble 24 hpf progenitors, with the exception of a few cells found at the end of the trajectory, while 24 hpf and 44 hpf differentiating cells highly overlap (Fig. 7B). These data further suggest transcriptional changes in early versus late hindbrain progenitors.

To identify the temporal cascade of TFs that may be involved in neurogenic cell-fate decision, we mapped TFs that significantly varied in their pseudo-temporal expression pattern, and clustered them according to their expression dynamic (Fig. 7C). This analysis highlights multiple discrete shifts in TF expression occurring during hindbrain neurogenesis. Seven distinct patterns were identified, where the first has high expression at the beginning of pseudotime, and the others present a progressive shift until reaching a peak of expression of neuronal markers at the end of differentiation. The first group (G1) includes *egr2b* and *mafba*, which are genes involved in segmental identity of progenitors that are rapidly downregulated at the onset of differentiation. In the next group (G2) are genes expressed in progenitors but not downregulated until later in pseudotime. These genes have been implicated in the maintenance of the progenitor fate and/or inhibition of neurogenesis. Among them, Zic and Her genes promote neural progenitor identity and inhibit differentiation (Bae et al., 2005; Coolen et al., 2012; Nyholm et al., 2007; Scholpp et al., 2009), Id genes encode negative regulators of proneural bHLH proteins and are abundant in multipotent cells (Ellis et al., 1990; Garrell and Modolell, 1990; Ling et al., 2014) and *zbtb16a* (*plzfa*) inhibits neurogenesis and the encoded protein is degraded in order for neuronal differentiation to progress (Sobieszczuk et al., 2010). The following group of genes (G3) with shifting expression in pseudotime are: *sox3*, which has initial constant expression followed by a drop in differentiated cells; *neurog1* (reviewed by Bertrand et al., 2002); *prdm12b*, a regulator of V1 interneuron fate decision (Thélie et al., 2015; Zannino et al., 2014); and *foxp4*, which is progressively expressed during neuronal differentiation and promotes detachment of differentiating cells from the neuroepithelium (Rousso et al., 2012). *atoh1b* and *neurod4* are found in the next step of the cascade (G4), together with *ebf2*, a factor that acts downstream of proneural genes and is necessary for initiation of migration toward the mantle layer and neuronal differentiation (Garcia-Dominguez et al., 2003). In the next group, a subset of genes initiates expression that then increases late in pseudotime (G5). They include *zbtb20*, which functions during corticogenesis as a temporal regulator for the generation of layer-specific neuronal subtypes (Tonchev et al., 2016), and the less studied *uncx*, *nhlh2*, *lhx4* and *sox12*. Furthermore, members of the zebrafish scratch family (*scrt1a/scrt1b/scrt2*) have a similar dynamic pattern and show enrichment within the neurogenic zone, with some dorsoventral differences: *scrt1a* and *scrt1b* are expressed ventrally and dorsally (Fig. 7D,E), while *scrt2* is only found ventrally (Fig. 7F). These genes have been implicated in the onset of neuronal migration (Itoh et al., 2013; Paul et al., 2014). Followed by a group of TFs with a later onset of expression that does not decline (G6), these factors are implicated in neuronal specification (*otpa*, *tal1* and *pax2a*). The final group of genes with an onset of expression late in pseudotime (G7) also encode regulators of neuronal identity (*isl1/isl2a*, *gata3* and *lhx1a/lhx5/lhx9*; Fig. S14).

To further explore the role of TFs in hindbrain neurogenesis, we used a complementary approach that does not relay on pseudotemporal ordering. A genetic regulatory network (GRN) was created using GENIE3 (Huynh-Thu et al., 2010), which uses a Random Forest machine-learning algorithm to predict the strength of putative regulatory links between a target gene and the expression pattern of input genes (i.e. transcription factors). As there have been extensive studies of gene regulation during hindbrain segmentation (Parker and Krumlauf, 2017), we tested whether GENIE3 finds known interactions. We analysed the transciptome data from 16 hpf and focussed on a module that includes regulators of segmentation and A-P identity (Fig. S15). We find potential interactions between *egr2b*, *mafba*, Hox genes and *epha4a*, which include seven interactions that have been verified *in vivo* (asterisks in Fig. S15). A GRN was produced for each individual stage (16 hpf Table S5.1, 24 hpf Table S5.2, 44 hpf Table S5.3), and we present findings for 44 hpf as these are more relevant for late steps of neurogenesis. To focus on the predictions with higher significance, we applied a threshold of >0.025 of important measure (IM) and these interactions were analysed in Cytoscape (Shannon et al., 2003) (Table S5). This cut-off recovered 4637 total interactions that constitute a valuable resource to guide future *in vivo* functional validations. Given the complexity of the network, we extracted a submodule to exemplify its predictive potential. We interrogated the network to specifically predict the role of Scrt genes during neurogenesis, and extracted their closest neighbours (Fig. 7G). This network module predicts interconnections between genes in G5a, G5a and G4. *scrt1a* and *scrt2* are found in a feedback loop with *nhlh2*, and upstream of neurogenic factors (*neurod4*, *elavl3*, *otpa/otpb* and *pax2a*), while *scrt1b* is connected to *atoh1a/atoh1b*, *atoh8* and *barhl1a/barhl1b*.

## DISCUSSION

The single cell transcriptome atlas that we present here is a resource for further investigation of mechanisms that regulate neurogenesis and other aspects of hindbrain development. We analysed the transcriptome of hindbrain cells prior to (16 hpf), during (24 hpf) and after (44 hpf) the patterning of neurogenesis to form discrete neurogenic and non-neurogenic zones within segments. We used unbiased methods to cluster cells based on transcriptional differences, and identified genes that mark distinct hindbrain segments, cell types along the D-V axis and neuronal differentiation. By comparing our findings with previous studies, we have created an annotated list of genes that indicates those that are previously known and those that are novel markers, as also highlighted in the relevant Results section.

Seurat analysis at 16 hpf clustered cells based on segment-specific gene expression and gave a global picture of differences in the transcriptome of distinct segments. The organisation of clusters from r2 to r6 suggests that neighbouring segments have a similar transcriptome, but with a significant difference between odd- and even-numbered segments. This is consistent with previous studies showing nested expression of Hox genes that regulate anterior-posterior identity (reviewed by Alexander et al., 2009; Tümpel et al., 2009), and the role of *egr2* in regulating gene expression in r3 and r5 that confers distinct properties from r2, r4 and r6 (Voiculescu et al., 2001). In contrast, r7 cells do not cluster adjacent to r6 cells, suggestive of a distinct identity that could indicate it is a transitional zone to the anterior spinal cord.

We find major differences in gene expression in differentiating neurons at 16 hpf and 24 hpf compared with 44 hpf, as expected from the generation of distinct neuronal cell types at different times. Our analyses reveal new genes that are co-expressed with known markers of neuronal cell types that form along the D-V axis. In addition to transcription factors, these include modulators of the Shh, RA and Wnt pathways. Interestingly, many differentiating neurons at all stages express *fstl1a*, suggesting a potential role of BMP inhibition. The generation of different neuronal cell types at 44 hpf compared with 16 hpf and 24 hpf is accompanied by changes in gene expression in progenitor cells at these stages, including proliferation markers and miR9 microRNAs. By carrying out pseudotime analysis, we inferred progressive changes in gene expression during the differentiation of progenitor cells to neurons. These data suggest a cascade in which genes that define segmental identity are rapidly downregulated, followed by factors that maintain progenitor cells, in turn followed by upregulation of genes required for neuronal migration and transcription factors that define neuronal identity. We also analysed transcription factor expression using an algorithm to predict gene regulatory networks. We focussed on Scrt family genes that regulate neuronal migration, and found potential relationships with proneural factors and regulators of neuronal identity. We envisage that investigators can interrogate the network for other TFs of interest to guide biological hypotheses and phenotypic screening of specific mutants.

One motivation for this study was to find genes that mark the distinct neurogenic and non-neurogenic zones that are established in the zebrafish hindbrain. These features are not found in the unbiased analysis, as this is dominated by the greatest transcriptomic differences. We therefore used known markers of hindbrain boundary cells, neurogenic cells and segment centres to drive clustering of the progenitor population. In addition, we carried out RNA-seq analyses after manipulation of Fgf pathway activation, which inhibits neurogenesis at segment centres. These analyses identified novel signalling factors, most notably follistatin and meteorin family members expressed in boundary cells and/or segment centres that are candidates to inhibit neurogenesis or promote gliogenesis. The single cell transcriptome data will enable investigators to extract information on other specific cell populations using this approach.

## MATERIALS AND METHODS

### Maintenance of zebrafish strains and husbandry

Zebrafish embryos were raised at 28.5°C or 25°C depending on the required stage (Westerfield, 2007). Embryos were staged according to hours post fertilisation (hpf) and morphological criteria (Kimmel et al., 1995). The zebrafish work was carried out under a UK Home Office Licence under the Animals (Scientific Procedures) Act 1986 and underwent a full ethical review.

### Mutant strains and heat shock treatment

*fgf20a* (dob) mutant embryos (Whitehead et al., 2005) were obtained from homozygous mutant in-crosses. Transgenic Tg(*hsp70:ca-fgfr1*) embryos are heterozygotes from outcrosses (Gonzalez-Quevedo et al., 2010; Marques et al., 2008). To induce constitutively active Fgfr1, Tg(*hsp70:ca-fgfr1*) embryos at 22 hpf were heat shocked for 30 min at 38.5°C and then incubated for 2 h at 28.5°C. Because around 50% of the embryos are carrying the transgene, controls and treated embryos were collected from the same heat-shocked clutch, avoiding any issue with differences in genomic background and changes in gene expression due to the heat-shock treatment. After mRNA extraction, qPCR was performed to identify properly dissected tissues and discriminate between controls and *fgfr1* overexpressing tissues.

### Whole-mount *in situ* hybridisation

For whole-mount *in situ* hybridisation, embryos or explants were fixed in 4% PFA overnight at 4°C, or for 4 h at room temperature, and kept in methanol at −20°C prior to processing. Some probes have been previously described: *neurog1* and *neurod4* (Alexander et al., 2009; Gonzalez-Quevedo et al., 2010), *pax2* (Krauss et al., 1991), *rfng* (Cheng et al., 2004), *etv5b* (cb805, ZFIN), *metrnla* (MPMGp609H2240Q8, RZPD) and *sox3* (EST clone: IMAGp998H108974Q). Additional probes were generated from cDNA of 20-44 hpf embryos. A forward primer was used together with a reverse primer with a T7 promoter site (5′gaaatTAATACGACTCACTATAGg3′) for amplification; see Table S6. Digoxigenin-UTP-labelled riboprobes were synthesised and *in situ* hybridisation performed as previously described (Xu et al., 1994). After BCIP/NBT colour development, embryos were re-fixed for 30 min, cleared in 70% glycerol/PBS, and mounted to view the dorsal or lateral side. For each gene at least two independent replicates were performed using more than 30 embryos each time. For transverse sections, embryos were extensively washed in PBST prior to mounting in 4% agarose/water. Embryos were sectioned using a Vibratome (Lecia VT1000 S), generating transverse sections of a thickness of 40 μm. Imaging was carried out with a Zeiss Axioplan2 with an Axiocam HRc camera.

### Hindbrain dissection

Embryos at the desired stage were dechorionated and de-yolked in DMEM with high Glucose, no Glutamine, no Calcium (11530556, Gibco); hindbrains were micro-dissected using 0.33 mm micro-fine sterile needles. Dissected tissues were kept in DMEM until further processed. For RNA-seq a single hindbrain tissue was collected in an individual tube and the quality of the dissection evaluated by qPCR (data not shown). For scRNA-seq, around 40 tissues per stage were pooled and immediately processed for cell dissociation.

### RNA extraction, cDNA preparation and qPCR

RNA was isolated using Quick-RNA Microprep kit (Zymo Research) and eluted in 15 μl (Lan et al., 2009). To evaluate the quality of dissection, 3 μl of RNA was reverse transcribed using SuperScript III Reverse Transcriptase (ThermoFisher Scientific), and the remainder stored at −80°C until processed. Primers for target genes were designed using PrimerQuest (IDT). qPCR was performed using QuantStudio 3 (ThermoFisher Scientific) with SYBR green Platinum SYBR Green qPCR SuperMix-UDG (ThermoFisher Scientific) master mix. The ΔΔCt method was used to calculate gene expression (Livak and Schmittgen, 2001). β-Actin was used as a reference gene. Primers used are listed in Table S7. Samples without contamination were processed for RNA-seq.

### Library preparation and RNA-sequencing

Libraries for the *fgf20a*^−/−^ experiment were prepared with the Ovation RNA-Seq System V2 (7102, NuGEN) for cDNA amplification, followed by NexteraXT (Illumina) for library preparation. These libraries were sequenced on the HiSeq 2000 (Illumina), with paired-end 75 bp reads. Libraries for the constitutive active Fgfr1 experiment were prepared with the Clontech SMARTer kit (634926, TaKaRa) for cDNA amplification, followed by NexteraXT (Illumina) for library preparation. These libraries were sequenced on the HiSeq 4000 (Illumina), with single-ended 75 bp reads.

### Sequence alignment and analysis of differentially expressed genes

The quality of the samples was assessed using FastQC. Reads were aligned against zebrafish genome GRCz10 and Ensembl release 89 transcript annotations using STAR v2.5.1b (Dobin et al., 2013) via the transcript quantification software RSEM v1.2.31 (Li and Dewey, 2011). Gene-level counts were rounded to integers and subsequently used for differential expression analysis with DESeq2 v1.20.0 (Anders and Huber, 2010) using default settings. Differential expression results were thresholded for significance based on an FDR≤0.01, a fold-change of ±2 and a minimum normalised count of >30 in all contributing samples from at least one of the replicate groups being compared. Heatmaps were created using rlog transformed count data, scaled across samples using a z-score.

### Preparation of single cells from zebrafish hindbrain

Around 40 hindbrain tissues per stage (16 hpf, 24 hpf and 44 hpf) were dissected as described above. The samples were incubated with FACS max cell dissociation solution (T200100, Amsbio) supplemented with 1 mg/ml Papain (10108014001, Sigma) for 25 min at 37°C and resuspended once during incubation. Cells were then transferred to HBSS (no calcium, no magnesium, no Phenol Red; 11140035, ThermoFisher Scientific) supplemented with 5%FBS, Rock inhibitor (Y-27632, Stem Cell Technologies) and 1× non-essential amino acids (11140035, ThermoFisher Scientific). Cells were further disaggregated by pipetting and filtered several times using 20 µm strainers (130-101-812, Miltenyi Biotech). To access quality live/cell death, cell size and number of clumps were measured. Samples with a viability above 65% were used for single cell sequencing. During protocol optimisation, qPCR was carried out to check that gene expression levels are similar in dissociated cells and the intact hindbrain.

### 10X genomics single-cell library preparation

A suspension of 10,000 single cells was loaded onto the 10X Genomics Single Cell 3′ Chip. cDNA synthesis and library construction were performed according to the manufacturer's protocol for the Chromium Single Cell 3′ v2 protocol (PN-120233, 10X Genomics). cDNA amplification involved 12 PCR cycles. Samples were sequenced on Illumina HiSeq 4000 using 100 bp paired-end runs.

### Bioinformatic analysis of scRNA-seq data

The 10X Cell Ranger software was used to de-multiplex Illumina BCL output, create fastq files and generate single cell feature counts for each library using a transcriptome built from the zebrafish Ensembl release 89, GRCz10.

### Seurat unsupervised analysis of aggregated data

Three 10X libraries representing the 16 hpf, 24 hpf and 44 hpf stages of embryonic development were aggregated using the 10X software ‘cellranger aggr’ function, which sub-samples reads such that all libraries have the same effective sequencing depth. Aggregated count data were further analysed using the Seurat v3.1.0 (Butler et al., 2018) package within R v3.6.1.

Cell quality was assessed using some simple QC metrics: library size, total number of expressed genes and mitochondrial RNA content. Outlier cells were flagged if they were above/below three median absolute deviations (MADs) from the median for any metric in a dataset-specific manner.

Data were normalised across cells using the ‘LogNormalize’ function with a scale factor of 10,000. A set of genes highly variable across cells was identified using the ‘FindVariableGenes’ function (selection.method=’vst’, nfeatures=2000). Data were centred and scaled using the ‘ScaleData’ function with default parameters.

PCA analysis was performed on the scaled data using the variant genes. Significant principal components were identified by manual inspection of the top loading genes and by plotting the standard deviations of the top 100 components.

The first 30 principal components were used to create a Shared Nearest Neighbour (SNN) graph using the ‘FindNeighbours’ function (k.param=20). This was used to find clusters of cells showing similar expression using the FindClusters function (resolution=0.8).

The Uniform Manifold Approximation and Projection (UMAP) dimensional reduction technique was used to visualise data from the first 30 principal components in two-dimensional space (‘RunUMAP’ function). Graphing of the output enabled visualisation of cell cluster identity and marker gene expression.

Visual inspection of hindbrain and non-hindbrain marker genes suggested some clusters were contaminated with non-hindbrain cells; see Table S1 for a list of valid hindbrain cells. A new iteration of the analysis was then performed as above, this time excluding contaminant cells from the aggregated data prior to normalisation, variable gene selection, data scaling, dimension reduction (PC1-30) and cluster identification (resolution=0.8).

Biomarkers of each cluster were identified using Wilcoxon rank sum tests using Seurat's ‘FindAllMarkers’ function. It was stipulated that genes must be present in 10% of the cells in a cluster and show a logFC of at least 0.25 to be considered for testing. Only positive markers were reported. The expression profile of top markers ranked by average logFC were visualised as heatmaps and dotplots of the scaled data. Cluster identity was determined using visual inspection focussing on the expression of known marker genes.

### Seurat unsupervised analysis of individual stages

Count data for individual stages were loaded directly into Seurat from the 10X results files separately, without aggregation. Downstream analysis was conducted as for the aggregated dataset. For each stage dataset, the first 30 principal components were used for cluster identification. Differing resolutions were passed to the ‘FindClusters’ function based on how well the resultant clusters corroborated known marker gene expression: 16 hpf (resolution=0.7), 24 hpf (resolution=1.2) and 44 hpf (resolution=1.0). The 16 hpf data were further analysed at higher resolution and also using PlotClusterTree in Seurat.

### Seurat supervised clustering of ventral progenitors from individual stages

For each stage, cells identified as being ventral progenitors in the aggregate analysis were subset and subjected to supervised clustering using custom sets of marker genes to drive PCA analysis, cluster identification and UMAP dimensional reduction. For 24 hpf ventral progenitor cells, the genes used were *rfng* (boundary), *etv5b* (segment centre), and *neurog1* and *neurod4* (neuronal differentiation). For 44 hpf ventral progenitor cells, the list was restricted to *etv5b*, *neurog1* and *neurod4*.

### Pseudotime analysis of aggregated dataset using Monocle3

Pseudotime analysis was conducted using the Bioconductor package Monocle v3.0.2 (Trapnell et al., 2014). Count data from the individual stages were combined. The ‘preprocess_cds’ function was used to normalise the data to address sequencing depth differences before PCA dimensional reduction (*n*=50). The three datasets were then aligned by fitting a linear model to the PCA coordinates of the cell and subtracting a ‘stage’ effect (‘align_cds’ function: num_dim=50, alignment_group=’stage’). Next, the data were subjected to UMAP dimensional reduction and cell clustering (‘cluster_cells’: resolution=0.001). A principal graph was plotted through the UMAP using the ‘learn_graph’ function, representing the path through development. The graph was in turn used to order cells through the developmental program as pseudotime using Sox3-positive 16 hpf cells at the start of the program.

Genes changing as a function of pseudotime were determined using graph-auto-correlation analysis (‘graph_test’ function). Selected genes listed as being transcription factors in the AnimalTFDB3.0 database were presented on a heatmap of expression over pseudotime.

### GENIE3 inference of regulatory networks

The Bioconductor package GENIE3 v1.4.3 (Huynh-Thu et al., 2010) was used to infer regulatory networks of genes within cells of individual developmental stages. For each stage, an expression matrix of raw gene counts, with non-hindbrain cells removed, was constructed and passed to the GENIE3 function together with a list of zebrafish transcription factors identified in the AnimalTFB3.0 database (targets=NULL, treeMethod=‘RF’, K=‘sqrt’, nTrees=1000) in order to create a weighted adjacency matrix. The weights describe the likelihood of a regulator-gene/target-gene link being genuine. This matrix was converted to a table of regulatory links (regulator-gene, target-gene and link-weight). Regulator/target links with weights >0.025 (data available in Table S5) were visualised as an interaction directed network within Cytoscape (Shannon et al., 2003).

## Supplementary Material

Supplementary information

Reviewer comments
