# Supplementary material for: A single cell transcriptome atlas of the developing zebrafish hindbrain
Source: Development. 2020 Mar 16;147(6):dev184143. doi: 10.1242/dev.184143 (PMC7097387; doi:10.1242/dev.184143)
Supplement: Supplementary information [file develop-147-184143-s1.pdf]

SUPPLEMENTARY FIGURES

Fig.S1

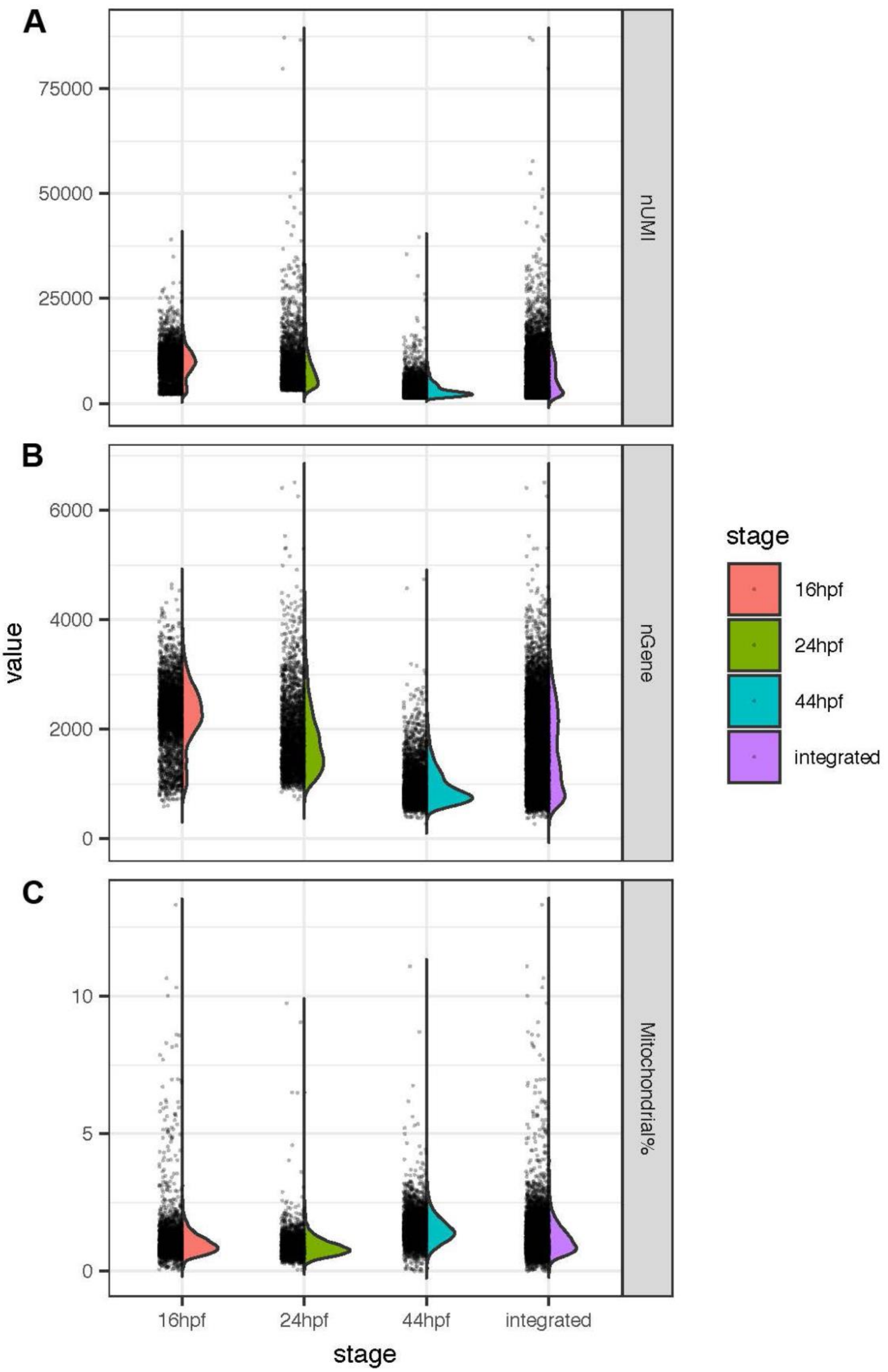

Development • Supplementary information

Figure S1. Quality Control matrix.

(A) Distribution of number of Unique Molecular Identifiers (UMIs), (B) number of genes per cell (nGene), and (C) percentage of mitochondrial reads are shown for 16 hpf (pink), 24 hpf (green), 44 hpf (blue) and the aggregated data set (violet).

Development • Supplementary information

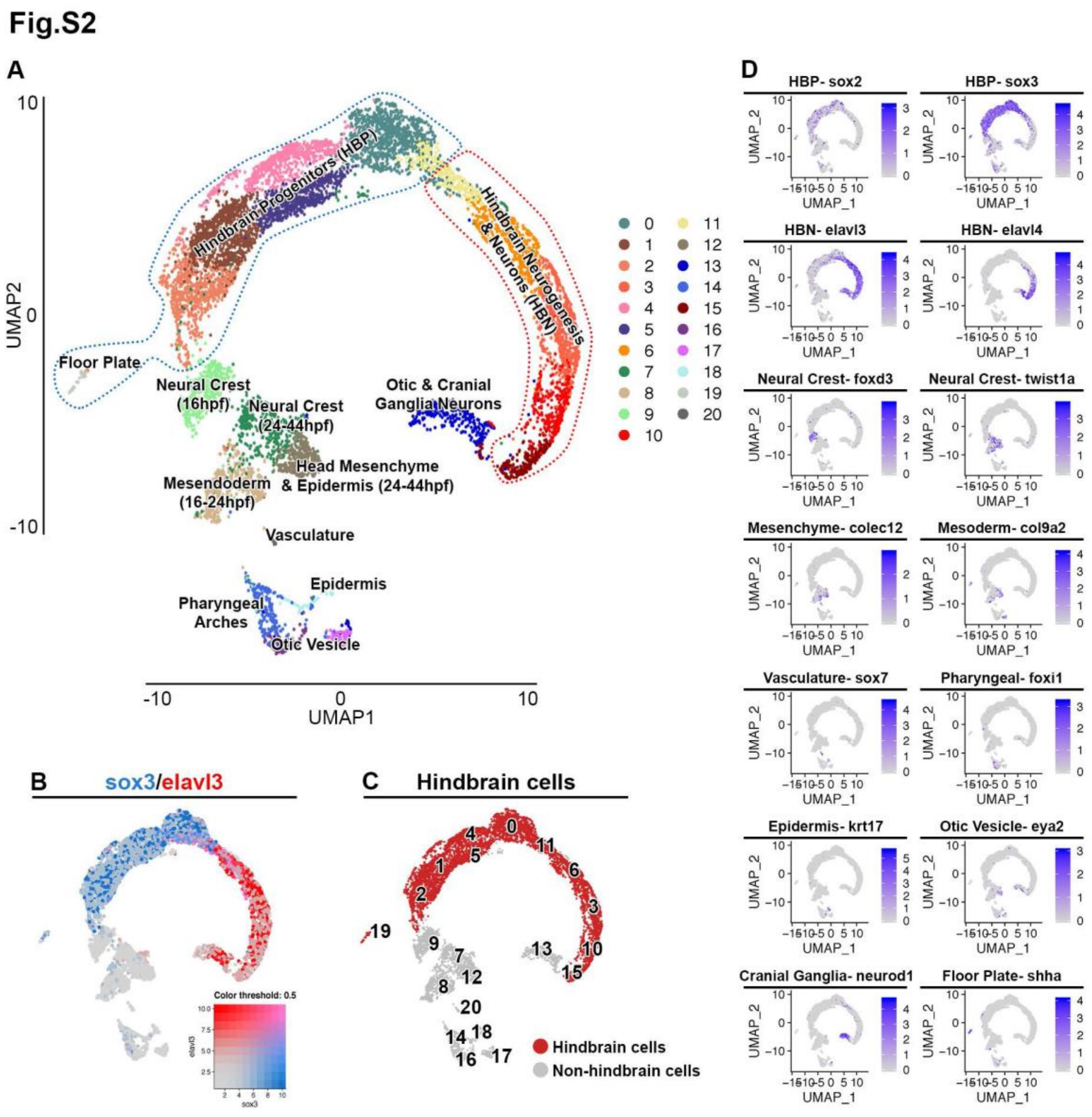

Figure S2. Mapping of the hindbrain and surrounding tissues.

(A) UMAP representation of the aggregated data (16 hpf, 24 hpf and 44 hpf), where the clustering of cells depicts their transcriptional similarity. (B) UMAP plot showing the expression distribution of *sox3* (blue) and *elavl3* (red). (C) High levels of *sox3* and/or *elavl3* expression demarcate the hindbrain territory. Cluster identity (A) was defined based on expression of known marker genes (*sox2, sox3* = hindbrain progenitors HBP; *elavl3, elavl4* = hindbrain neurons HBN; *foxd3*, *twis1* = neural crest; *colec12* = head mesenchyme; *col9a2* = mesoderm; *sox7* = vasculature; *foxi1* = pharyngeal arches; *krt17* = epidermis; *eya2* = otic vesicle; *neurod1* = cranial ganglia; *shha* = floor plate) (D). Colour intensity is proportional to the expression level of a given gene.

Development • Supplementary information

Fig.S3

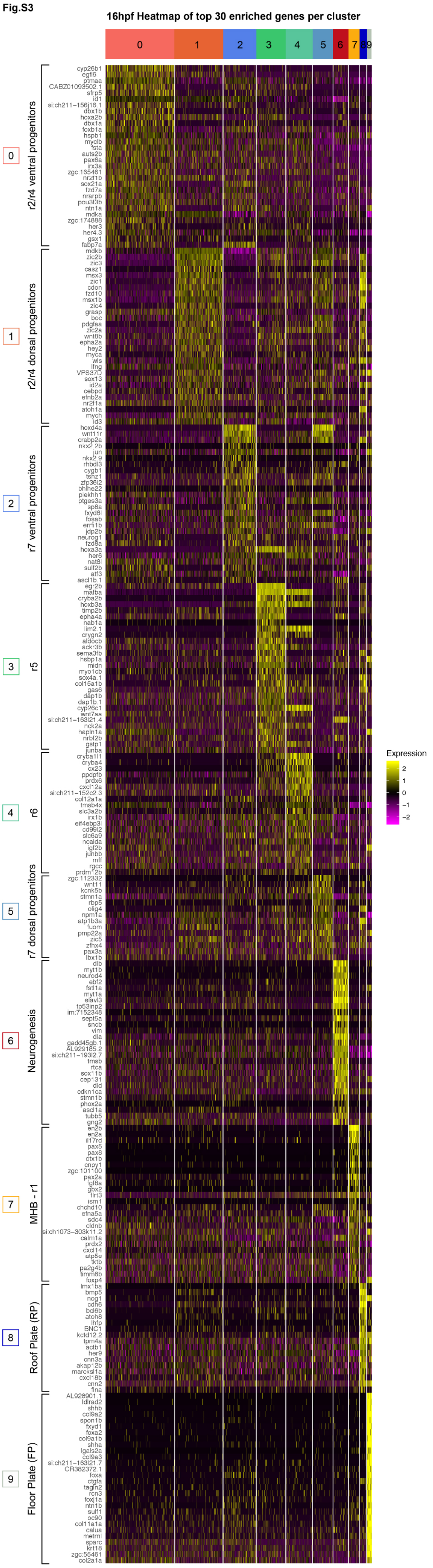

Development • Supplementary information

Figure S3. Heatmap of the top 30 significant markers per cluster at 16 hpf.

Full heatmap of the top 30 significant markers per cluster, if available, at 16 hpf.

Development • Supplementary information

Fig.S4

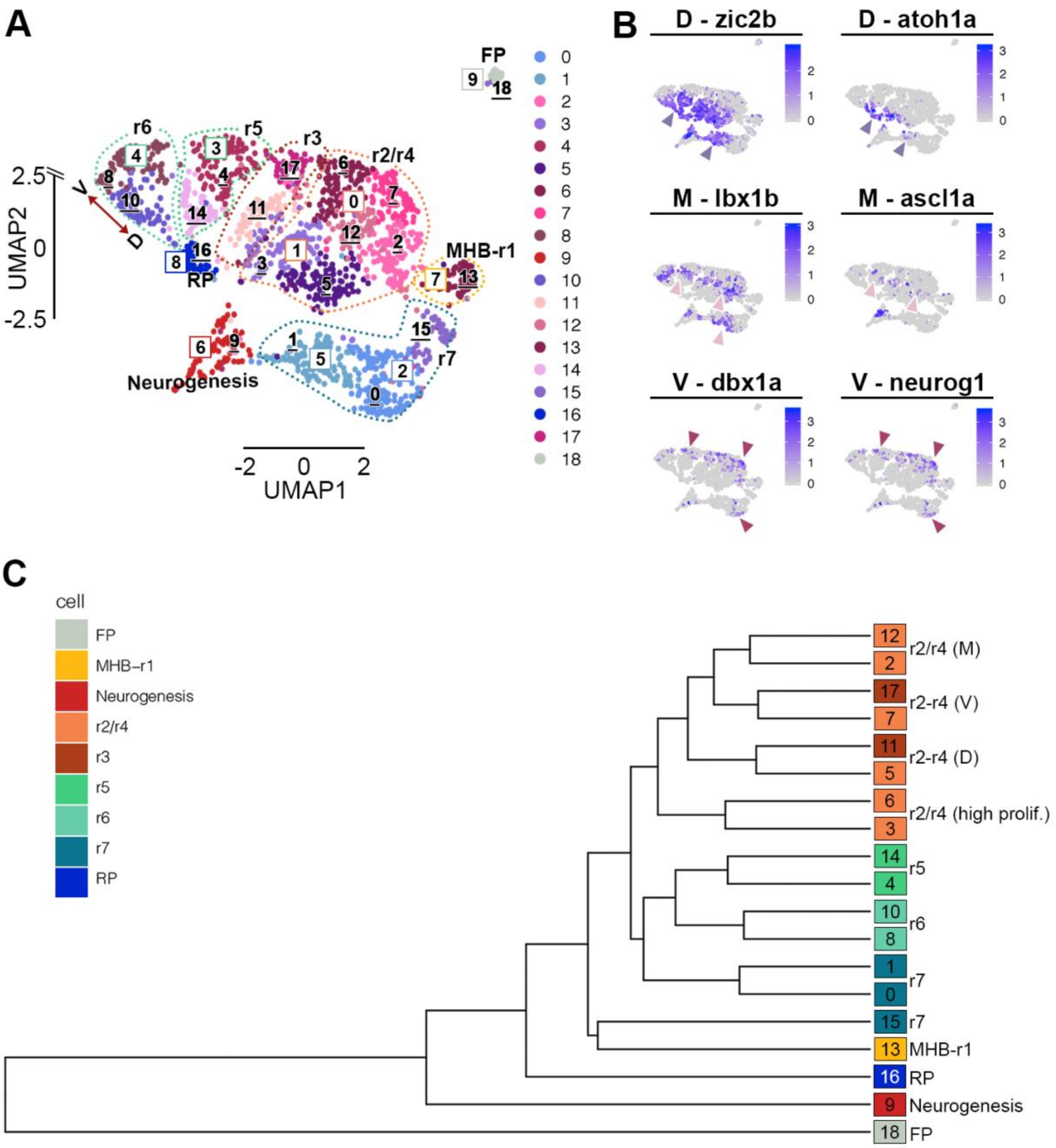

Figure S4. Subclustering of 16 hpf hindbrain.

(A) Higher resolution analysis of the 16 hpf hindbrain cells identifies 19 clusters. Dorsal, medial and ventral progenitors are separated in distinct clusters along the anterior-posterior axis. Clusters identified in Fig. 2A are overlaid to visualize rhombomere identity. r3 is now separated from r2/r4, and multiple clusters appear in the r2/r4 domain. UMAP2 (y-axis) is discontinuous. (B) Dorsal (*zic2b, atoh1a*), medial (*lbx1b, ascl1a*) and ventral (*dbx1a, neurog1*) gene expression domains are reported. Colour intensity is proportional to the expression level of a given gene. (C) Analysis with PlotClusterTree in Seurat to reveal the transcriptomic similarities between clusters. Cluster identity in (A) and (C) are colour coded as indicated.

Fig.S5

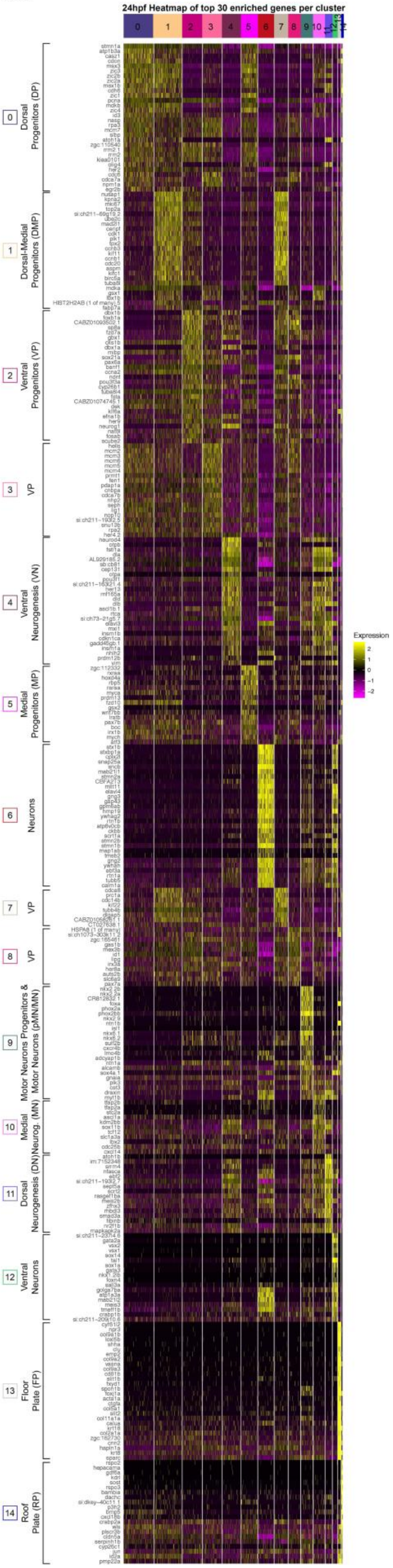

Development • Supplementary information

Figure S5. Heatmap of the top 30 significant markers per cluster at 24 hpf.
Full heatmap of the top 30 significant markers per cluster, if available, at 24 hpf.

Development • Supplementary information

Fig.S6

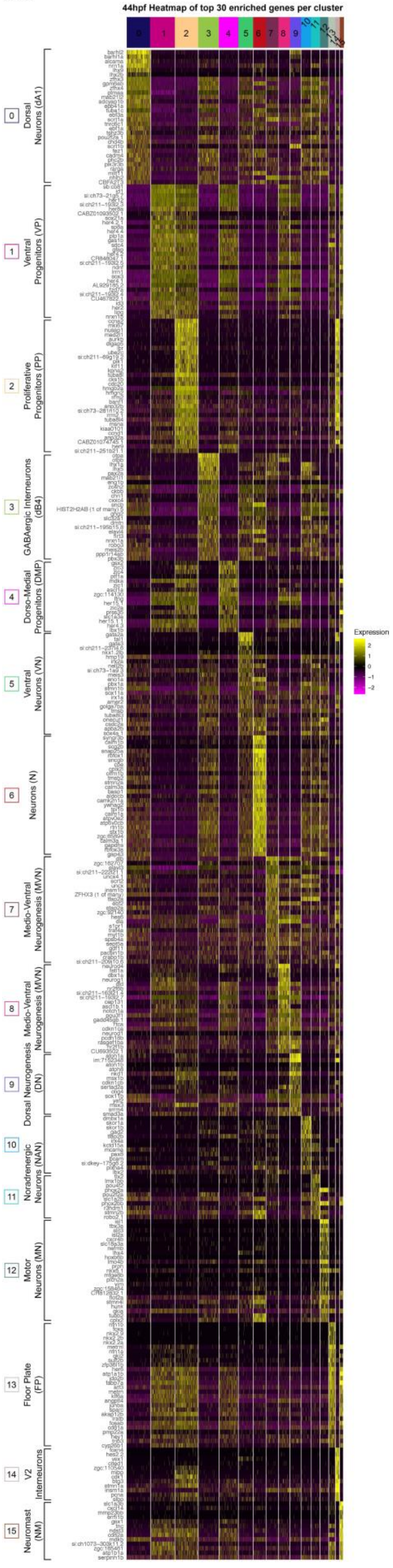

Development • Supplementary information

Figure S6. Heatmap of the top 30 significant markers per cluster at 44 hpf.

Full heatmap of the top 30 significant markers per cluster, if available, at 44 hpf.

Development • Supplementary information

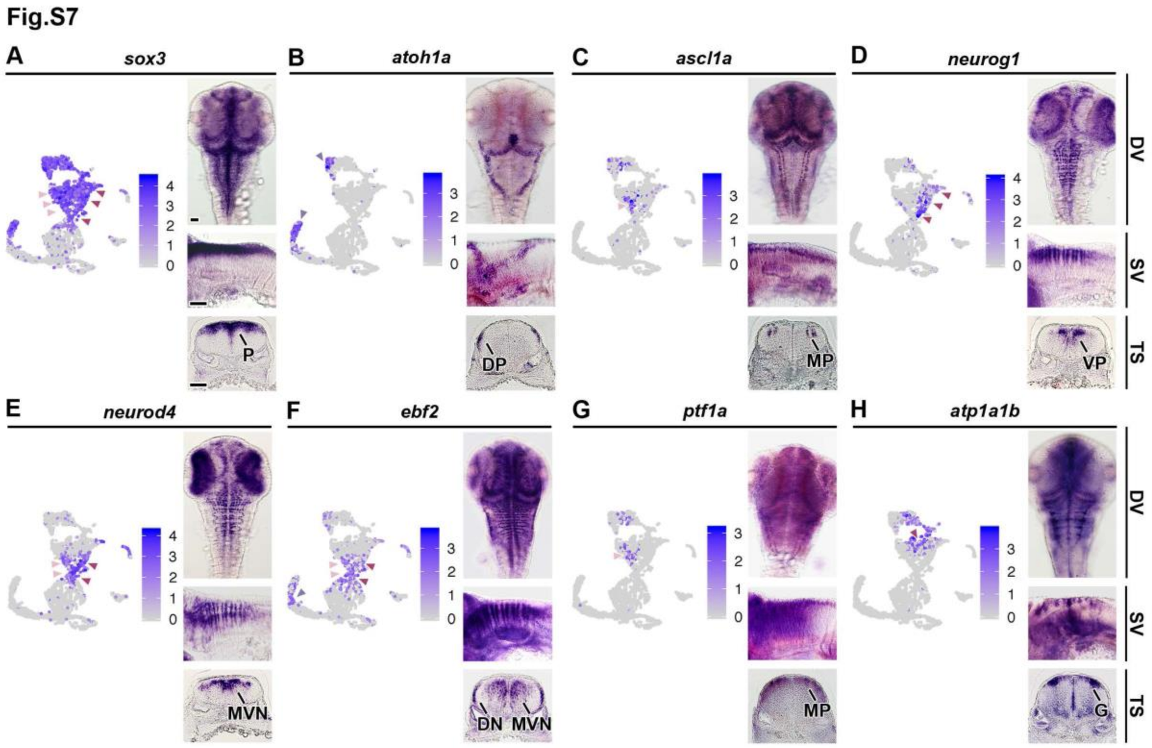

Figure S7. Selected expression patterns of progenitors and differentiation factors at 44 hpf.

Progenitor marker *sox3* (A) is widely expressed in the ventricular zone. Proneural genes *atoh1a* (B), ascl1a (C) and *neurog1* (D) are differentially expressed along the D-V axis at 44 hpf, similarly to their distribution at 24 hpf. *neurod4* (E) is found in medio-ventral differentiating progenitors, *ebf2* (F) has an expression domain resembling *neurod4*, and also expressed in some differentiated neurons, while *ptf1a* (G) is found medially in differentiating cells. *atp1a1b* is a newly identified marker of glial cells (H). For each gene the UMAP plot shows gene expression from the 44 hpf scRNA-seq data; colour intensity is proportional to the expression level of a given gene. In situ hybridization images are shown for dorsal view (DV), side view (SV) and transverse section (TS) at the level of r4-r5/r5-r6. scRNA-seq and in situ hybridization expression patterns strongly correlate. P = Progenitors, DP = Dorsal Progenitors, MP = Medial Progenitors, VP = Ventral Progenitors, MVN = Medio-Ventral Neurogenesis, DN = Dorsal Neurogenesis, G = Glia.

Development • Supplementary information

Fig.S8

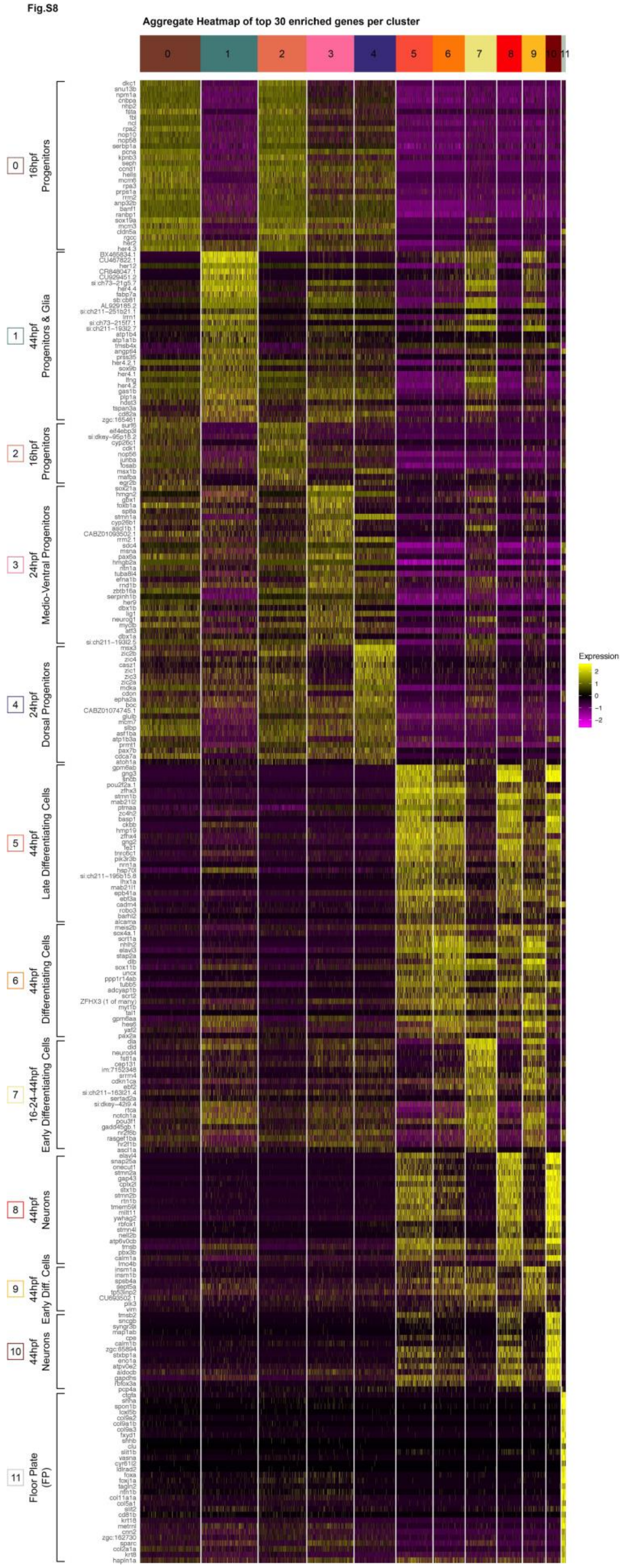

Development • Supplementary information

Figure S8. Heatmap of the top 30 significant markers per cluster for the aggregate data set.

Full heatmap of the top 30 significant markers per cluster, if available, for the aggregate data set.

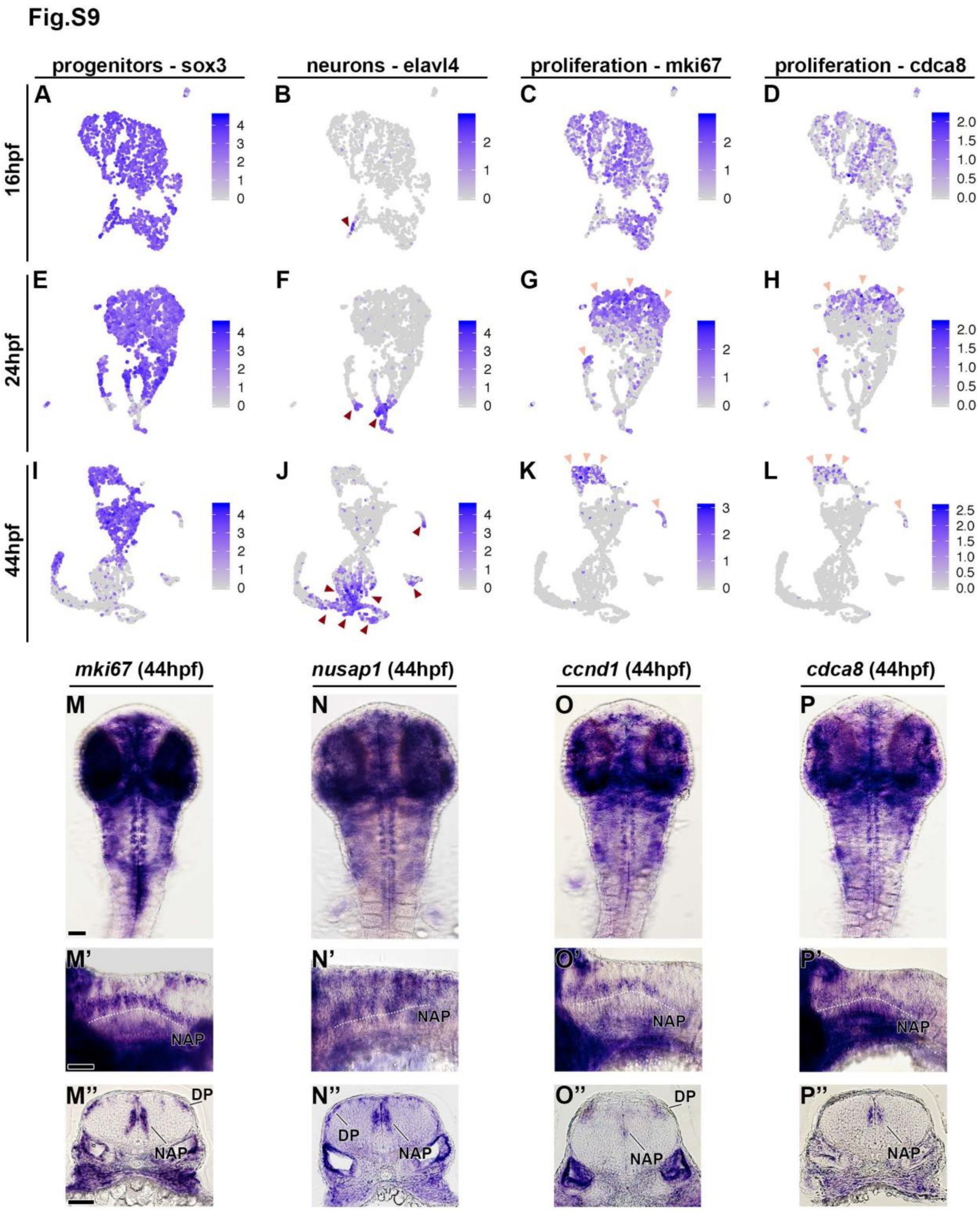

Figure S9. Progenitor, neurogenesis and proliferation gene expression at different stages.

For each gene, the UMAP plot shows gene expression from 16 hpf (A-D), 24 hpf (E-H) and 44 hpf (I-L) scRNA-seq data. Progenitor cells are marked by *sox3* expression (A, E, I), neurogenesis by *elavl4* (B, F, J) and proliferation by *mki67* (C, G, K) and *cdca8* (D, H, L). Whole mount in situ hybridization at 44 hpf of *mki67* (M), *nusap1* (N), *ccnd1* (O) and *cdca8*

Development • Supplementary information

(P). Dorsal view (M-P), side view (M'-P') and 40 µm hindbrain transverse section at the level of r4-r5/r5-r6 (M''-P''). Scale bar: 50 µm.

Fig.S10

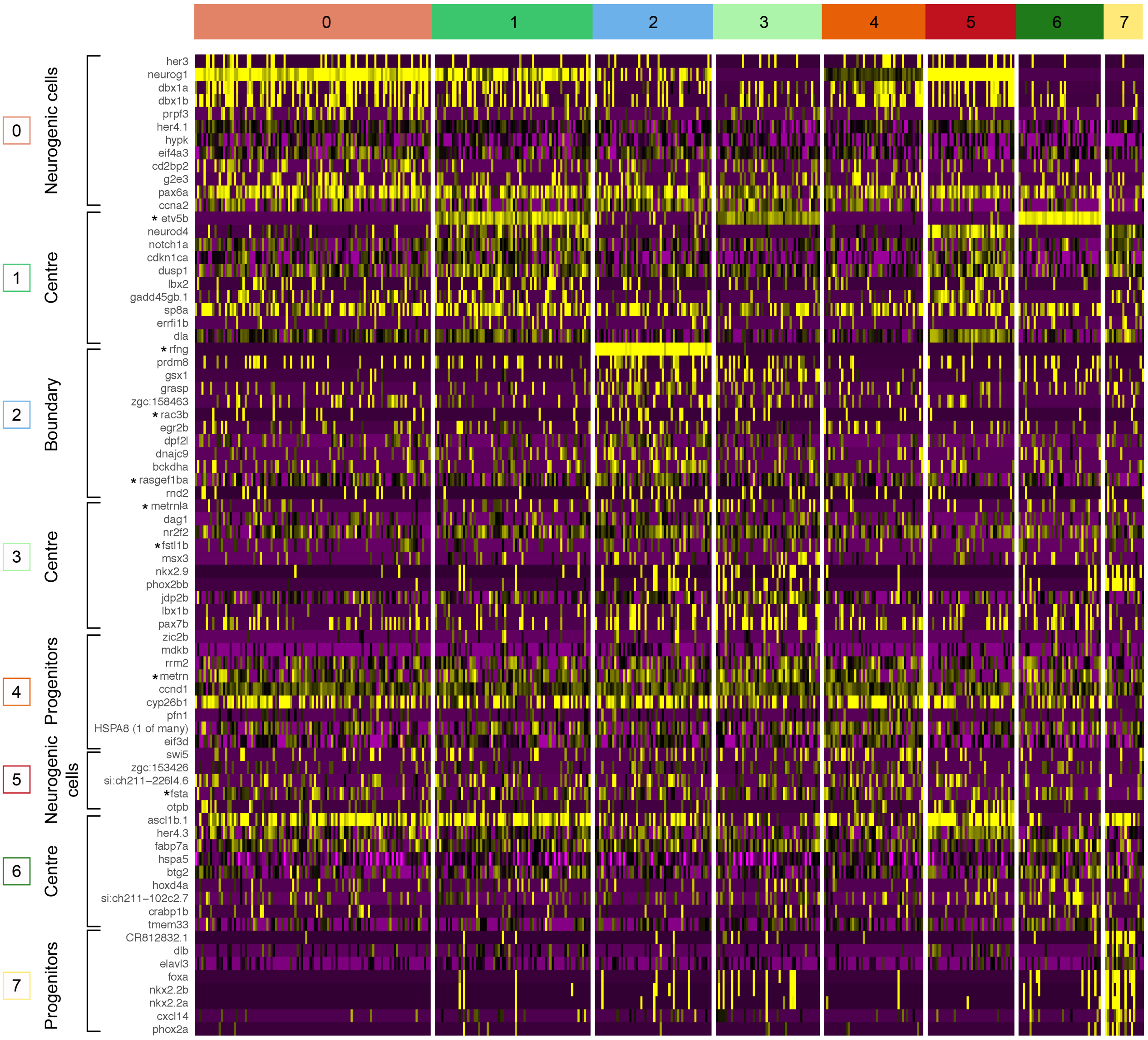

Figure S10. Selected top markers for 24 hpf boundary and centre supervised analysis. Heatmap of selected markers (pval<0.1, logfc>0.1, detected at a minimum fraction of 20% of tested cells) of the supervised clustering analysis done on 24 hpf ventral progenitors (VP). Validated and known makers of boundary and rhombomere centre cells are highlighted with an asterisk.

Fig.S11

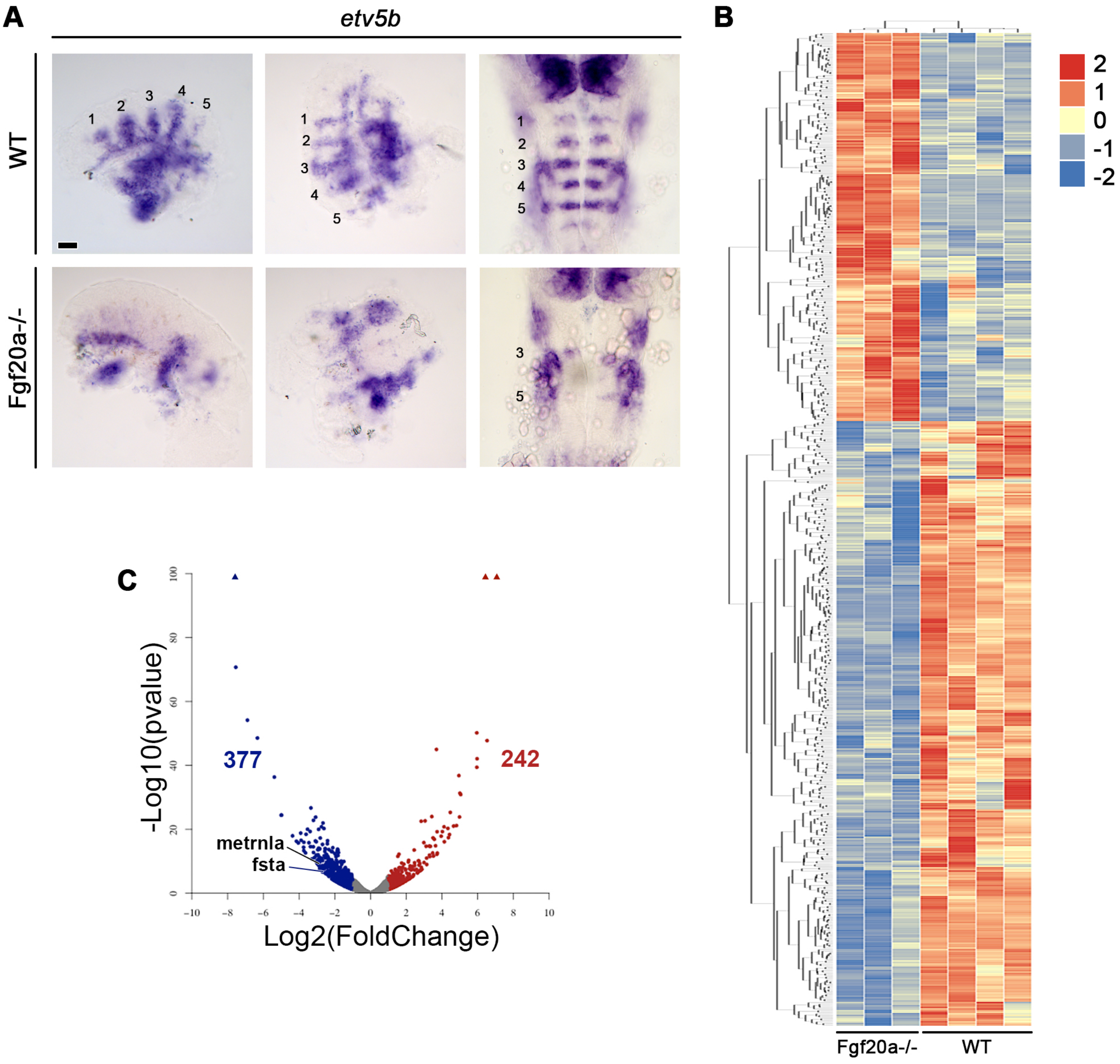

Figure S11. Fgf20a-/- bulk RNA-seq identifies metrnl and fsta as new Fgf20 targets in the hindbrain.

(A) Examples of *etv5b* expression in dissected hindbrain for wild-type (WT) and *fgf20a-/-* 24 hpf embryos. Five stripes of segment centre expression occur in WT embryos, together with otic vesicle and cranial ganglia expression domains. In *fgf20a-/-* embryos only weak r3 and r5 stripes are present, while otic vesicle and cranial ganglia expression domains are unaffected. Representative whole embryos are also shown. Strong expression in domains outside the hindbrain probably masks changes in the hindbrain (e.g. *etv5b*). Scale bar: 50 µm. (B) Heatmap showing RNA-seq expression levels of significantly differentially expressed genes between 4 WT and 3 *fgf20a-/-* dissected tissues. Hierarchical clustering groups the WT tissues and the mutants in separate clusters, suggesting genome wide

Development • Supplementary information

similarities in dissected samples of the same genotype. Colour scale depicts low to high expression in blue to red shades, respectively. (C) Volcano plot shows 377 significantly downregulated genes in blue and 242 upregulated in red. *metrnl* and *fsta* are among the downregulated factors. Grey dots are non-significant genes, x-axis Log2(Fold Change) and y-axis -Log10(pvalue).

Development • Supplementary information

Fig.S12

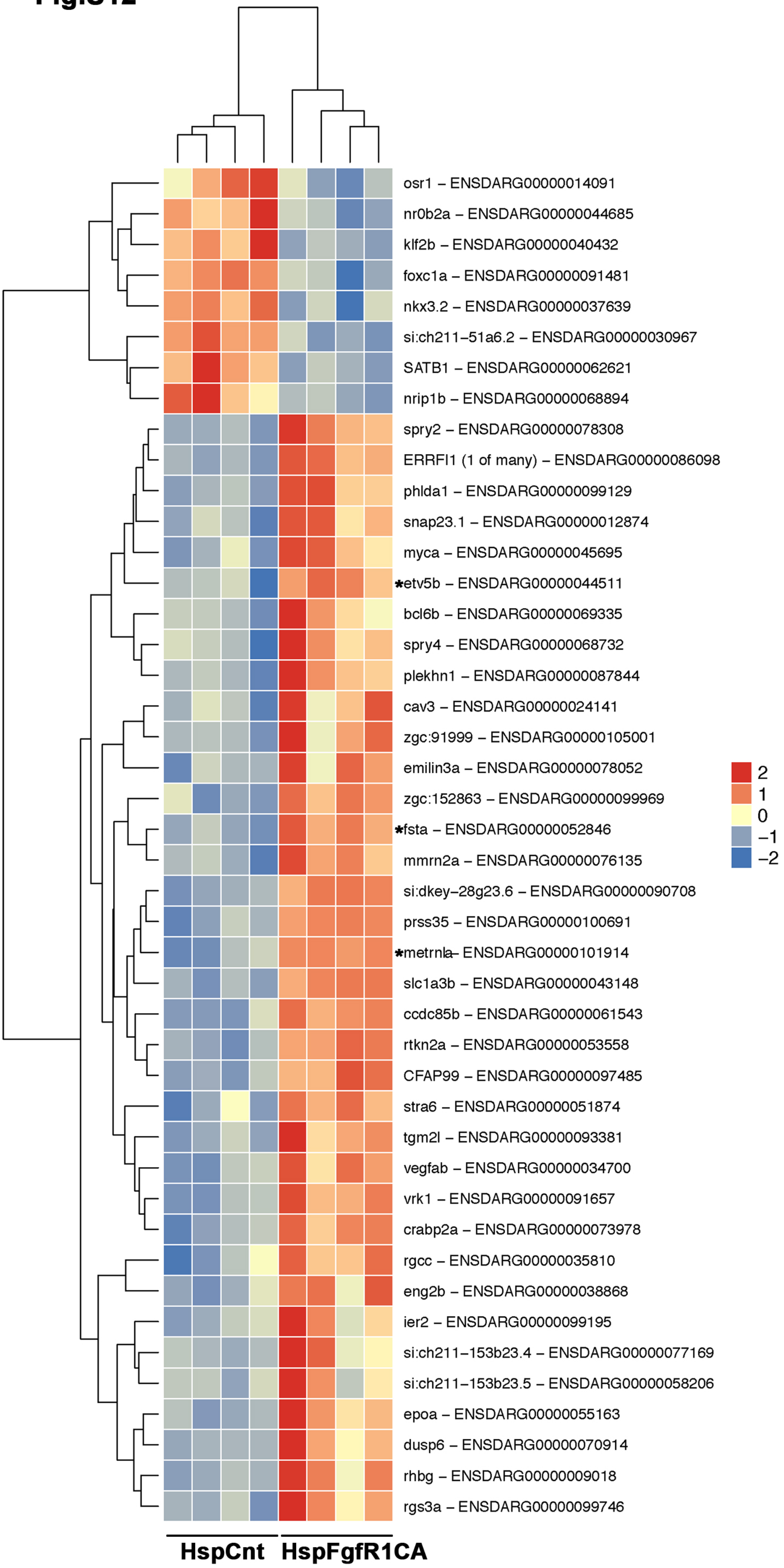

Development • Supplementary information

Figure S12. Constitutive activation of FgfR1 ectopically induces *etv5b, metrnl* and *fsta* expression.

Heatmap shows RNA-seq expression levels of significantly differentially expressed genes between 4 heat shocked controls (HspCnt) and 4 heat shocked constitutive active FgfR1 (HspFgfR1CA) dissected tissues. Hierarchical clustering groups the 4 HspCnt tissues and the 4 HspFgfR1CA in separate clusters, suggesting genome wide similarities in dissected samples of the same genotype. Colours scale depicts low to high expression in blue to red shades, respectively. 8 genes are significantly downregulated in HspFgfR1CA, while 36 are upregulated. Among the upregulated genes, known Fgf signaling targets are found (e.g. *spry2, spry4* and *etv5b*) and in addition *metrnl* and *fsta* are found that are expressed in hindbrain segment centres.

Fig. S13

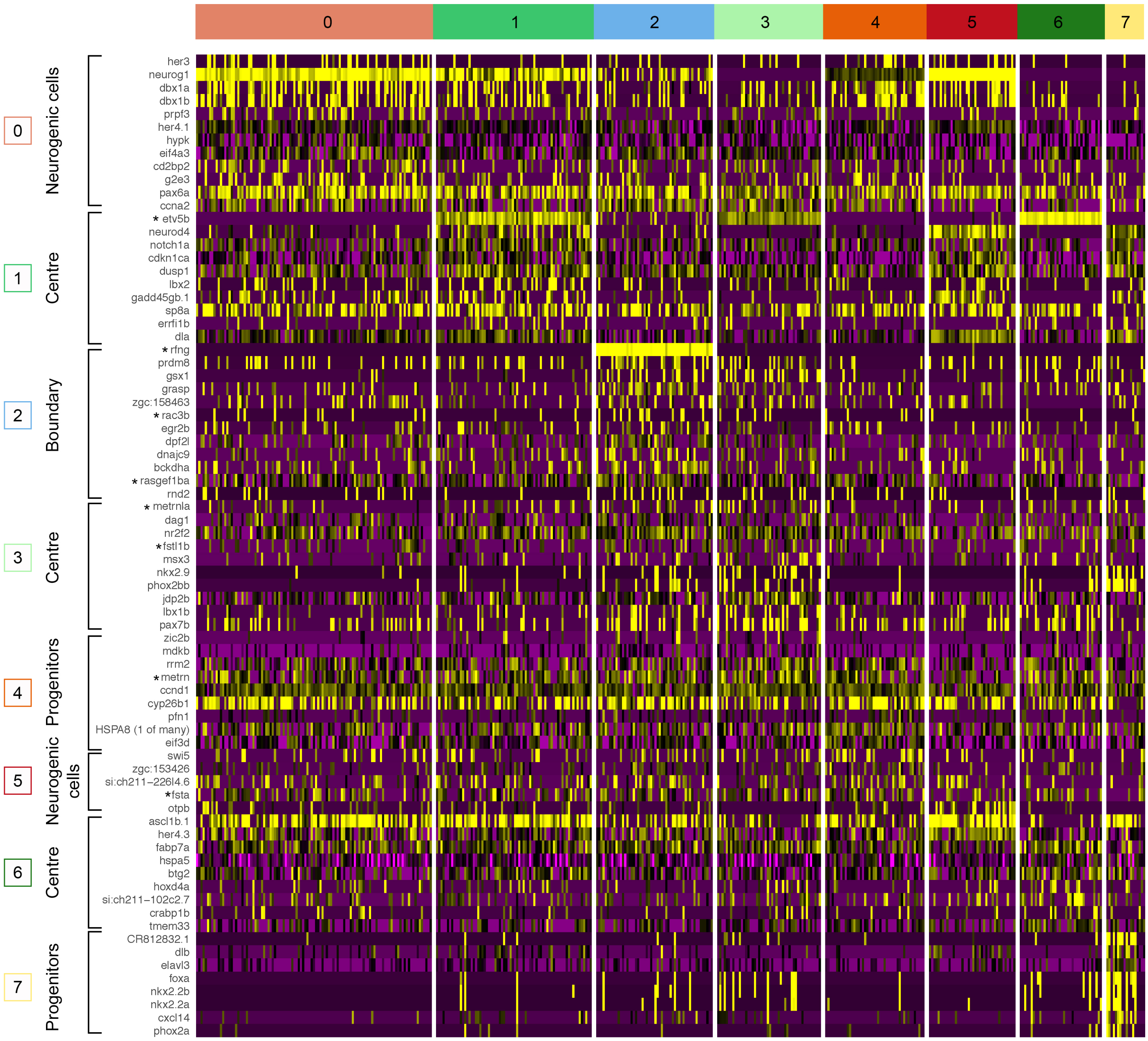

Development • Supplementary information

Figure S13. Heatmap of the top 15 significant markers per cluster for 44 hpf centre progenitors supervised analysis.

Full heatmap of the top 15 significant markers per cluster, if available, for 44 hpf centre progenitors supervised analysis.

Development • Supplementary information

Fig.S14

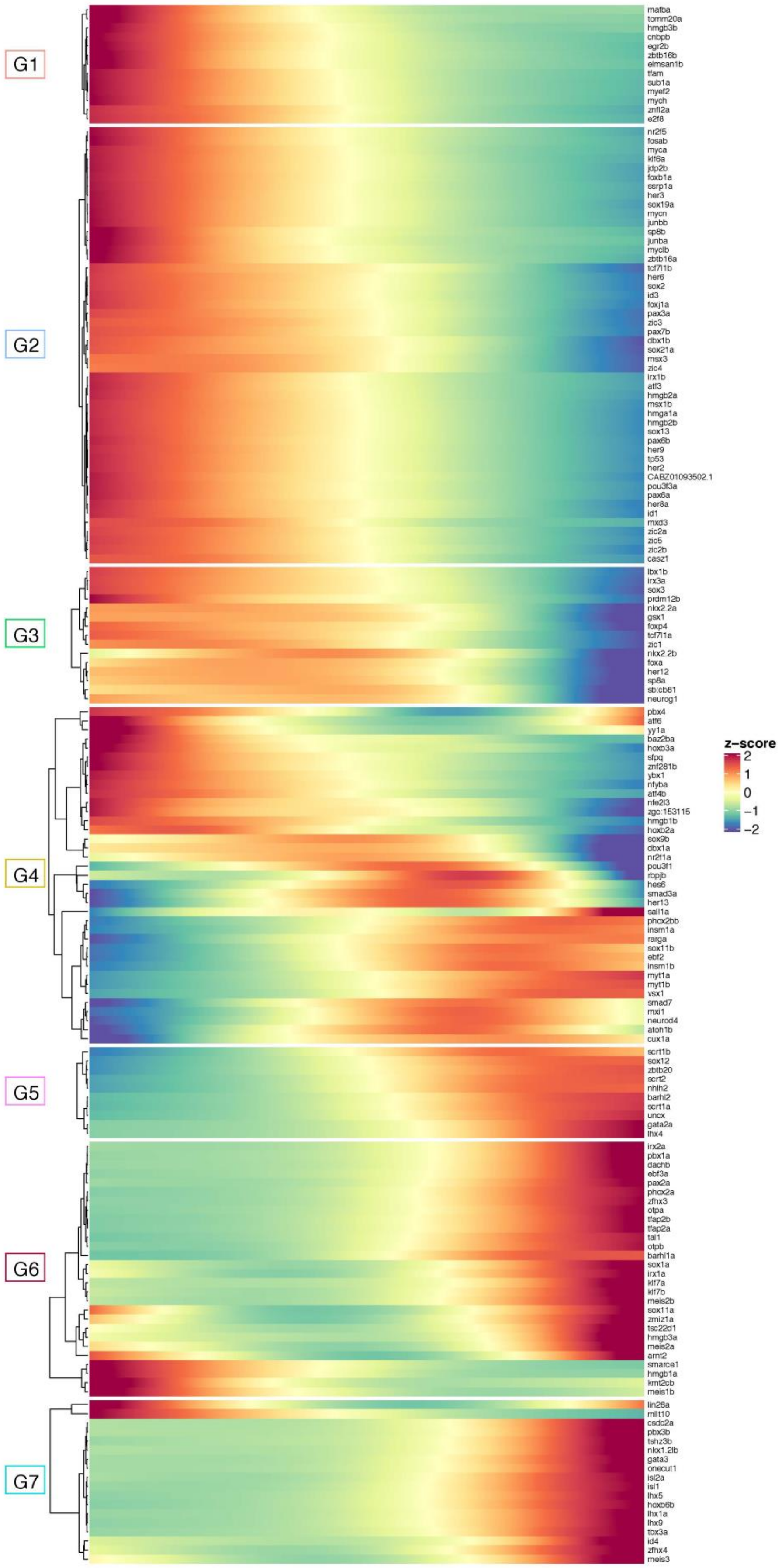

Development • Supplementary information

Figure S14. Heatmap of selected transcription factors changing with pseudotime.

Full heatmap of selected transcription factors changing along the pseudo-temporal axis.

Development • Supplementary information

Fig.S15

A

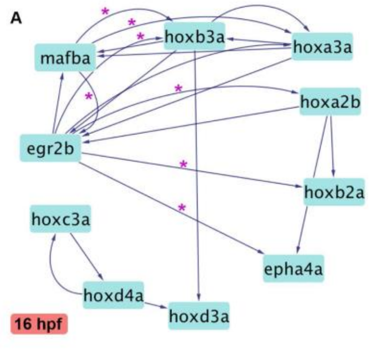

B

Regulatory Gene	Target Gene	Weight	Refs
mafba	egr2b	0.0617	Chomette et al., 2006; Labalette et al., 2011
egr2b	hoxa2b	0.0632	Nonchev et al., 1996a; Nonchev et al., 1996b
egr2b	hoxb2a	0.0469	Sham et al., 1993; Nonchev et al., 1996a
mafba	hoxa3a	0.0983	Manzanares et al., 1999; Manzanares et al., 1997
hoxb3a	hoxa3a	0.0875	Manzanares et al., 2001
mafba	hoxb3a	0.1841	Manzanares et al., 1999; Manzanares et al., 1997; Manzanares et al., 2002
egr2b	hoxb3a	0.0380	Manzanares et al., 2002
egr2b	epha4a	0.1782	Theil et al., 1998

Figure S15. Known interactions retrieved with Genie3 at 16 hpf.

(A) Interaction among *egr2b* (*krox20*), *hox* genes (*hoxa2b, hoxb2a, hoxa3a, hoxb3a, hoxc3a, hoxd3a, hoxd4a*) and *mafba* from the predicted gene regulatory network. * marks known interaction that have been validated in vivo. (B) Table summarizing the retrieved known interactions with relative weight predicted by Genie3 interaction and references of the correspondent in vivo validations.

Development • Supplementary information

SUPPLEMENTARY TABLES

Table S1. Hindbrain cells.

Spreadsheet containing the names of cells considered to be hindbrain on the basis of marker gene expression.

Click here to Download Table S1

Table S2. Expression pattern summary.

Spreadsheet 1 - Table S2.1. Expression pattern summary of selected genes differentially expressed at 16 hpf.

Spreadsheet 2 - Table S2.2. Expression pattern summary of selected genes differentially expressed at 24 hpf.

Spreadsheet 3 - Table S2.3. Expression pattern summary of selected genes differentially expressed at 44 hpf.

Spreadsheet 4 - Table S2.4. Expression pattern summary of selected genes differentially expressed in the Aggregate data set.

Spreadsheet 5 - Table S2.5. Expression pattern summary of differentially expressed genes between boundary and centre progenitors at 24 hpf.

Spreadsheet 6 - Table S2.6. Expression pattern summary of enriched genes in centre progenitors at 44 hpf.

References are listed below.

Click here to Download Table S2

Table S3. Differential expression of significant genes between wild-type and Fgf20a-/-.

Bulk RNA-seq analysis of 4 wild-type (WT) and 3 Fgf20a-/- dissected hindbrain tissues.

Click here to Download Table S3

Table 4. Differential expression of significant genes between heat shock controls and HspFgfR1CA.

Bulk RNA-seq analysis of 4 heat shock control (HspCnt) and 4 heat shock constitutive active FgfR1 (HspCAFgfR1) dissected hindbrain tissues.

Click here to Download Table S4

Table S5. GENIE3 predicted interactions (IM>0.025)

Genie3 table of interactions presenting regulatoryGene, targetGene and weight of the interaction (IM>=0.025).

Spreadsheet 1 - Table S5.1. Predicted interaction at 16hpf

Spreadsheet 2 - Table S5.2. Predicted interaction at 24hpf

Spreadsheet 3 - Table S5.3. Predicted interaction at 44hpf

Click here to Download Table S5

Development • Supplementary information

Table S6. Primer sequences for antisense probe generation

Gene	Primer sequences 5′ to 3′
atoh1a	Fw CCAACGTCGTGCAGAAA<br>Rw gaaatTAATACGACTCACTATAGgAACCCATTACAAAGCCCAGATA
ascl1a	Fw CAAAGAGCCAAGGGACTAAGAG<br>Rw gaaatTAATACGACTCACTATAGgCCCAGCATTGTAAAGGCAAAG
barhl2	Fw GCCACCTCCTCCTTTCTAATC<br>Rw gaaatTAATACGACTCACTATAGgGCTGTCCACGGTTCCTAATAA
otpb	Fw CTCACGGGCTCATACAACTATT<br>Rw gaaatTAATACGACTCACTATAGgGACGCAGGTGTCAACAATTTAG
tal1	Fw GCGGAACAGTATGGGATGTAT<br>Rw gaaatTAATACGACTCACTATAGgCTGGAATGGTGTAGTCCTCTTG
cldn5a	Fw AGCAGACAACCTGACCAAAG<br>Rw gaaatTAATACGACTCACTATAGgTGGCACAAGCACGAAGAT
fstl1a	Fw CCGCCGTACCATTGAGAAA<br>Rw gaaatTAATACGACTCACTATAGgAGCAGTGTGGTCATCCTTTAC
mki67	Fw AGCCAGAAGATGCCAAACTTA<br>Rw gaaatTAATACGACTCACTATAGgGGACTACCTCACCAGCACTAAAC
fabp7a	Fw GCAATGTTACCAAACCCACAAT<br>Rw gaaatTAATACGACTCACTATAGgACAAAGGCAGGCCTCAATAA
atp1b4	Fw GCCATGTTTGCTGGTTGTATG<br>Rw gaaatTAATACGACTCACTATAGgGTGTCGTGTTGGACGTTAAGA
CU929451.2	Fw TGCCTCAGCAGTGTCTAAAG<br>Rw gaaatTAATACGACTCACTATAGgTCAGACACATTTGGTAGCTTCA
rac3b	Fw CAATGTGATGGTGGATGGTAAAC<br>Rw gaaatTAATACGACTCACTATAGgACCCAACCTGTGAGAGTAGTA
fstl1b	Fw CAGTCCAGTCGTGTGTTATGT<br>Rw gaaatTAATACGACTCACTATAGgTGTGCTGGTCTTCATCTTCTC
fsta	Fw CTGTGGTCCTGGAAAGAGATG<br>Rw gaaatTAATACGACTCACTATAGgGACTCATCTTTGCATCCCATAAAC
plp1a	Fw ATGCTCTGCCTTCAGCTTATC<br>Rw gaaatTAATACGACTCACTATAGgCATGGAAACCAACCCTCTCTAC
her4.4	Fw CCGCCGTACCATTGAGAAA<br>Rw gaaatTAATACGACTCACTATAGgAGCAGTGTGGTCATCCTTTAC
rtca	Fw GCTGAAATGGCACCTCAAATAG<br>Rw gaaatTAATACGACTCACTATAGgCCTGTTCGCATTCTGGATGTA
dusp1	Fw CTGAGGTGATCTTGCCAGTATT<br>Rw gaaatTAATACGACTCACTATAGgGACAATCCCTGAGCAACCTATAA
zbtb18	Fw ATCCACCTCAGCACACATTT<br>Rw gaaatTAATACGACTCACTATAGgCCCACTCTTACCTTCACCTTTC
ebf2	Fw GTCATGGGTCTCAGCTCTTATC<br>Rw gaaatTAATACGACTCACTATAGgTGGCAACCTCCTCACAATC
atp1a1b	Fw GACCATCCCATCACTGCTAAA<br>Rw gaaatTAATACGACTCACTATAGgCCTCGTACGCCAGAGAAATAG
ptf1a	Fw CACAGGCTTAGACTCTTTCTCC<br>Rw gaaatTAATACGACTCACTATAGgCCCGTAGTCTGGGTCATTTG
prdm8	Fw TCGCTCCTTGTGGACTAATG<br>Rw gaaatTAATACGACTCACTATAGgCTGGCTTCTGTTGGTTGATTG
nusap1	Fw AACTGTCCTCACCACCAATAAA<br>Rw gaaatTAATACGACTCACTATAGgGACAAACGAGACGAAAGCTAAAC
ccnd1	Fw CGAGCTCCAGCTTTCTTACTT<br>Rw gaaatTAATACGACTCACTATAGgGCCAGATCCCACTTCAGTTTAT
Cdc8a	Fw CACCGCTGAAGTCTACAATGA<br>Rw gaaatTAATACGACTCACTATAGgGACGGGTACAGCACAAGAATA

Development • Supplementary information

Table S7. qPCR primers

Gene	Primer sequences 5′ to 3′	Species	Marker Region
β-Actin	Fw CGAGCTGTCTTCCCATCCA<br>Rw TCACCAACGTAGCTGTCTTT	Zebrafish	Housekeeping gene
otx2	Fw CAAGCAACCACCTTACACGG<br>Rw TCGTCTCTGCTTTCGAGGAG	Zebrafish	Anterior head
egr2b (*krox20*)	Fw GGACATTACGAGCAGATAAACG<br>Rw CTGCTGGAGTAGGCTAAGTCG	Zebrafish	Hindbrain
mafba	Fw AGCGTTTGATGGATACAGGG<br>Rw TGGTGTTGATGGTGATGGTG	Zebrafish	Hindbrain
hoxb2a	Fw CAGAGATTCAAGGTGGACTCG<br>Rw AGTAGCTGCGTGTTGGTATAC	Zebrafish	Hindbrain
etv5b	Fw CTCTTTCAAGACCTCAGCCAG<br>Rw GCTCATCTCCCTCTTTATTTTCG	Zebrafish	Hindbrain, FGF readout
hoxb6a	Fw GGGAAAAGCATCTACCCTGA<br>Rw CGACCAGCGTTACCGAAG	Zebrafish	Spinal Cord
xFgfR1	Fw CTGCTCTATCAGTTGCCCG<br>Rw CCCAGTTGATGCTCTGAACA	Xenopus	Heat Shock Tg(hsp70:ca-fgfr1)

Development • Supplementary information

SUPPLEMENTARY REFERENCES

Allende, M. L., et al. (1994). The expression pattern of two zebrafish achaete-scute homolog (ash) genes is altered in the embryonic brain of the cyclops mutant. *Dev. Biol.* **166**, 509-30.
Amoyel, M., et al. (2005). Wnt1 regulates neurogenesis and mediates lateral inhibition of boundary cell specification in the zebrafish hindbrain. *Development* **132**, 775-85.
Ando, H., et al. (2005). Lhx2 mediates the activity of Six3 in zebrafish forebrain growth. *Dev. Biol.* **287**, 456-68.
Appel, B., et al. (1995). Motoneuron fate specification revealed by patterned LIM homeobox gene expression in embryonic zebrafish. *Development* **121**, 4117-25.
Bae, Y. K., et al. (2005). Patterning of proneuronal and inter-proneuronal domains by hairy- and enhancer of split-related genes in zebrafish neuroectoderm. *Development* **132**, 1375-85.
Beretta, C. A., et al. (2011). All four zebrafish Wnt7 genes are expressed during early brain development. *Gene Expr Patterns* **11**, 277-84.
Cadwallader, A. B., et al. (2006). Combinatorial expression patterns of heparan sulfate sulfotransferases in zebrafish: I. The 3-O-sulfotransferase family. *Dev. Dyn.* **235**, 3423-31.
Caron, A., et al. (2012). Wnt/beta-catenin signaling directly regulates Foxj1 expression and ciliogenesis in zebrafish Kupffer's vesicle. *Development* **139**, 514-24.
Cheng, C. W., et al. (2007). Zebrafish homologue irx1a is required for the differentiation of serotonergic neurons. *Dev. Dyn.* **236**, 2661-7.
Cheng, Y. C., et al. (2004). Notch activation regulates the segregation and differentiation of rhombomere boundary cells in the zebrafish hindbrain. *Dev. Cell* **6**, 539-50.
Choe, S. K., et al. (2011). A screen for hoxb1-regulated genes identifies ppp1r14al as a regulator of the rhombomere 4 Fgf-signaling center. *Dev. Biol.* **358**, 356-67.
Chomette, D., et al. (2006). Krox20 hindbrain cis-regulatory landscape: interplay between multiple long-range initiation and autoregulatory elements. *Development* **133**, 1253-62.
Colombo, A., et al. (2006). Zebrafish BarH-like genes define discrete neural domains in the early embryo. *Gene Expr Patterns* **6**, 347-52.
Dee, C. T., et al. (2008). Sox3 regulates both neural fate and differentiation in the zebrafish ectoderm. *Dev. Biol.* **320**, 289-301.
Del Giacco, L., et al. (2006). Differential regulation of the zebrafish orthopedia 1 gene during fate determination of diencephalic neurons. *BMC Dev. Biol.* **6**, 50.
Duncan, R. N., et al. (2015). Identification of Wnt Genes Expressed in Neural Progenitor Zones during Zebrafish Brain Development. *PLoS One* **10**, e0145810.
Elsen, G. E., et al. (2008). Zic1 and Zic4 regulate zebrafish roof plate specification and hindbrain ventricle morphogenesis. *Dev. Biol.* **314**, 376-92.
Elsen, G. E., et al. (2009). The autism susceptibility gene met regulates zebrafish cerebellar development and facial motor neuron migration. *Dev. Biol.* **335**, 78-92.
Emoto, Y., et al. (2005). Retinoic acid-metabolizing enzyme Cyp26a1 is essential for determining territories of hindbrain and spinal cord in zebrafish. *Dev. Biol.* **278**, 415-27.
Esain, V., et al. (2010). FGF-receptor signalling controls neural cell diversity in the zebrafish hindbrain by regulating olig2 and sox9. *Development* **137**, 33-42.
Filipek-Gorniok, B., et al. (2015). The NDST gene family in zebrafish: role of NDST1B in pharyngeal arch formation. *PLoS One* **10**, e0119040.

Filippi, A., et al. (2005). The basic helix-loop-helix olig3 establishes the neural plate boundary of the trunk and is necessary for development of the dorsal spinal cord. *Proc. Natl. Acad. Sci. U. S. A.* **102**, 4377-82.
Fjose, A., et al. (1994). Expression of the zebrafish gene hlx-1 in the prechordal plate and during CNS development. *Development* **120**, 71-81.
Ghosh, P., et al. (2018). Analysis of novel caudal hindbrain genes reveals different regulatory logic for gene expression in rhombomere 4 versus 5/6 in embryonic zebrafish. *Neural Dev* **13**, 13.
Gorsi, B., et al. (2010). Dynamic expression patterns of 6-O endosulfatases during zebrafish development suggest a subfunctionalisation event for sulf2. *Dev. Dyn.* **239**, 3312-23.
Grinblat, Y., et al. (2001). zic Gene expression marks anteroposterior pattern in the presumptive neurectoderm of the zebrafish gastrula. *Dev. Dyn.* **222**, 688-93.
Gu, X., et al. (2005). Molecular cloning and expression of a novel CYP26 gene (cyp26d1) during zebrafish early development. *Gene Expr Patterns* **5**, 733-9.
Guner, B., et al. (2007). Cloning of zebrafish nkx6.2 and a comprehensive analysis of the conserved transcriptional response to Hedgehog/Gli signaling in the zebrafish neural tube. *Gene Expr Patterns* **7**, 596-605.
Gupta, M., et al. (2013). Identification and expression analysis of zebrafish glypicans during embryonic development. *PLoS One* **8**, e80824.
Haddon, C., et al. (1998). Multiple delta genes and lateral inhibition in zebrafish primary neurogenesis. *Development* **125**, 359-70.
Hans, S., et al. (2004). her3, a zebrafish member of the hairy-E(spl) family, is repressed by Notch signalling. *Development* **131**, 2957-69.
Hirate, Y., et al. (2006). Canopy1, a novel regulator of FGF signaling around the midbrain-hindbrain boundary in zebrafish. *Curr. Biol.* **16**, 421-7.
Hong, E., et al. (2013). Cholinergic left-right asymmetry in the habenulo-interpeduncular pathway. *Proc. Natl. Acad. Sci. U. S. A.* **110**, 21171-6.
Hsu, L. S., et al. (2010). Zebrafish calcium/calmodulin-dependent protein kinase II (cam-kii) inhibitors: expression patterns and their roles in zebrafish brain development. *Dev. Dyn.* **239**, 3098-105.
Kani, S., et al. (2010). Proneural gene-linked neurogenesis in zebrafish cerebellum. *Dev. Biol.* **343**, 1-17.
Kelly, G. M., et al. (1995). Zebrafish wnt8 and wnt8b share a common activity but are involved in distinct developmental pathways. *Development* **121**, 1787-99.
Kinkhabwala, A., et al. (2011). A structural and functional ground plan for neurons in the hindbrain of zebrafish. *Proc. Natl. Acad. Sci. U. S. A.* **108**, 1164-9.
Knight, R. D., et al. (2005). AP2-dependent signals from the ectoderm regulate craniofacial development in the zebrafish embryo. *Development* **132**, 3127-38.
Kondrychyn, I., et al. (2017). Transcriptional Complexity and Distinct Expression Patterns of auts2 Paralogs in Danio rerio. *G3 (Bethesda)* **7**, 2577-2593.
Korzh, V., et al. (1998). Expression of zebrafish bHLH genes ngn1 and nrd defines distinct stages of neural differentiation. *Dev. Dyn.* **213**, 92-104.
Kotkamp, K., et al. (2014). Pou5f1/Oct4 promotes cell survival via direct activation of mych expression during zebrafish gastrulation. *PLoS One* **9**, e92356.
Koudijs, M. J., et al. (2005). The zebrafish mutants dre, uki, and lep encode negative regulators of the hedgehog signaling pathway. *PLoS Genet* **1**, e19.
Kudoh, T., et al. (2001). A gene expression screen in zebrafish embryogenesis. *Genome Res.* **11**, 1979-87.
Labalette, C., et al. (2011). Hindbrain patterning requires fine-tuning of early krox20 transcription by Sprouty 4. *Development* **138**, 317-26.
Lee, H. C., et al. (2017). Embryonic expression patterns of Eukaryotic EndoU ribonuclease family gene endouC in zebrafish. *Gene Expr Patterns* **25-26**, 66-70.
Lee, S. A., et al. (2003). The zebrafish forkhead transcription factor Foxi1 specifies epibranchial placode-derived sensory neurons. *Development* **130**, 2669-79.

Li, J., et al. (2014). Temporal and spatial expression of the four Igf ligands and two Igf type 1 receptors in zebrafish during early embryonic development. *Gene Expr Patterns* **15**, 104-11.
Li, Y., et al. (2018). Temporal and spatial expression of fgfbp genes in zebrafish. *Gene* **659**, 128-136.
Liu, Q., et al. (2006). cadherin-6 message expression in the nervous system of developing zebrafish. *Dev. Dyn.* **235**, 272-8.
Love, C. E., et al. (2012). Expression and retinoic acid regulation of the zebrafish nr2f orphan nuclear receptor genes. *Dev. Dyn.* **241**, 1603-15.
Lun, K., et al. (1998). A series of no isthmus (noi) alleles of the zebrafish pax2.1 gene reveals multiple signaling events in development of the midbrain-hindbrain boundary. *Development* **125**, 3049-62.
Luo, N., et al. (2016). Syndecan-4 modulates the proliferation of neural cells and the formation of CaP axons during zebrafish embryonic neurogenesis. *Sci. Rep.* **6**, 25300.
Manzanares, M., et al. (2001). Independent regulation of initiation and maintenance phases of Hoxa3 expression in the vertebrate hindbrain involve auto- and cross-regulatory mechanisms. *Development* **128**, 3595-607.
Manzanares, M., et al. (1999). Conserved and distinct roles of kreisler in regulation of the paralogous Hoxa3 and Hoxb3 genes. *Development* **126**, 759-69.
Manzanares, M., et al. (1997). Segmental regulation of Hoxb-3 by kreisler. *Nature* **387**, 191-5.
Manzanares, M., et al. (2002). Krox20 and kreisler co-operate in the transcriptional control of segmental expression of Hoxb3 in the developing hindbrain. *EMBO J.* **21**, 365-76.
Mckeown, K. A., et al. (2012). Disruption of Eaat2b, a glutamate transporter, results in abnormal motor behaviors in developing zebrafish. *Dev. Biol.* **362**, 162-71.
Melvin, V. S., et al. (2013). A morpholino-based screen to identify novel genes involved in craniofacial morphogenesis. *Dev. Dyn.* **242**, 817-31.
Minchin, J. E., et al. (2008). Sequential actions of Pax3 and Pax7 drive xanthophore development in zebrafish neural crest. *Dev. Biol.* **317**, 508-22.
Miyake, A., et al. (2012). Neucrin, a novel secreted antagonist of canonical Wnt signaling, plays roles in developing neural tissues in zebrafish. *Mech. Dev.* **128**, 577-90.
Moens, C. B., et al. (1998). Equivalence in the genetic control of hindbrain segmentation in fish and mouse. *Development* **125**, 381-91.
Moens, C. B., et al. (1996). valentino: a zebrafish gene required for normal hindbrain segmentation. *Development* **122**, 3981-90.
Nakaya, N., et al. (2007). Expression patterns of alternative transcripts of the zebrafish olfactomedin 1 genes. *Gene Expr Patterns* **7**, 723-9.
Nechiporuk, A., et al. (2007). Specification of epibranchial placodes in zebrafish. *Development* **134**, 611-23.
Nepal, C., et al. (2016). Transcriptional, post-transcriptional and chromatin-associated regulation of pri-miRNAs, pre-miRNAs and moRNAs. *Nucleic Acids Res.* **44**, 3070-81.
Nikaido, M., et al. (2013). A systematic survey of expression and function of zebrafish frizzled genes. *PLoS One* **8**, e54833.
Nikolaou, N., et al. (2009). Lunatic fringe promotes the lateral inhibition of neurogenesis. *Development* **136**, 2523-33.
Nonchev, S., et al. (1996a). The conserved role of Krox-20 in directing Hox gene expression during vertebrate hindbrain segmentation. *Proc. Natl. Acad. Sci. U. S. A.* **93**, 9339-45.
Nonchev, S., et al. (1996b). Segmental expression of Hoxa-2 in the hindbrain is directly regulated by Krox-20. *Development* **122**, 543-54.
Ochi, H., et al. (2009). Lbx2 regulates formation of myofibrils. *BMC Dev. Biol.* **9**, 13.
Oxtoby, E., et al. (1993). Cloning of the zebrafish krox-20 gene (krx-20) and its expression during hindbrain development. *Nucleic Acids Res.* **21**, 1087-95.

Pauls, S., et al. (2012). Lens development depends on a pair of highly conserved Sox21 regulatory elements. *Dev. Biol.* **365**, 310-8.

Piotrowski, T., et al. (2000). The endoderm plays an important role in patterning the segmented pharyngeal region in zebrafish (Danio rerio). *Dev. Biol.* **225**, 339-56.

Prince, V. E., et al. (1998). Zebrafish hox genes: expression in the hindbrain region of wild-type and mutants of the segmentation gene, valentino. *Development* **125**, 393-406.

Radomska, K. J., et al. (2016). Characterization and Expression of the Zebrafish qki Paralogs. *PLoS One* **11**, e0146155.

Rauch, G. J., Lyons, D.A., Middendorf, I., Friedlander, B., Arana, N., Reyes, T., and Talbot, W.S. (2003). Submission and Curation of Gene Expression Data. ZFIN Direct Data Submission (http://zfin.org).

Riley, B. B., et al. (2004). Rhombomere boundaries are Wnt signaling centers that regulate metameric patterning in the zebrafish hindbrain. *Dev. Dyn.* **231**, 278-91.

Rohrschneider, M. R., et al. (2007). Zebrafish Hoxb1a regulates multiple downstream genes including prickle1b. *Dev. Biol.* **309**, 358-72.

Selland, L. G., et al. (2018). Coordinate regulation of retinoic acid synthesis by pbx genes and fibroblast growth factor signaling by hoxb1b is required for hindbrain patterning and development. *Mech. Dev.* **150**, 28-41.

Sham, M. H., et al. (1993). The zinc finger gene Krox20 regulates HoxB2 (Hox2.8) during hindbrain segmentation. *Cell* **72**, 183-96.

Sun, Z., et al. (2008). Discovery and characterization of three novel synuclein genes in zebrafish. *Dev. Dyn.* **237**, 2490-5.

Tanaka, H., et al. (2007). Novel mutations affecting axon guidance in zebrafish and a role for plexin signalling in the guidance of trigeminal and facial nerve axons. *Development* **134**, 3259-69.

Tendeng, C., et al. (2006). Cloning and embryonic expression of five distinct sfrp genes in the zebrafish Danio rerio. *Gene Expr Patterns* **6**, 761-71.

Theil, T., et al. (1998). Segmental expression of the EphA4 (Sek-1) receptor tyrosine kinase in the hindbrain is under direct transcriptional control of Krox-20. *Development* **125**, 443-52.

Thisse, B., Pflumio, S., Fürthauer, M., Loppin, B., Heyer, V., Degrave, A., Woehl, R., Lux, A., Steffan, T., Charbonnier, X.Q. And Thisse, C. (2001). Expression of the zebrafish genome during embryogenesis (NIH R01 RR15402). ZFIN Direct Data Submission (http://zfin.org).

Thisse, B., Thisse, C. (2004). Fast Release Clones: A High Throughput Expression Analysis. ZFIN Direct Data Submission (http://zfin.org).

Thisse, B., Wright, G.J., Thisse, C. (2008). Embryonic and Larval Expression Patterns from a Large Scale Screening for Novel Low Affinity Extracellular Protein Interactions. ZFIN Direct Data Submission (http://zfin.org).

Thisse, C., And Thisse, B. (2005). High Throughput Expression Analysis of ZF-Models Consortium Clones. ZFIN Direct Data Submission (http://zfin.org).

Thompson, M. A., et al. (1998). The cloche and spadetail genes differentially affect hematopoiesis and vasculogenesis. *Dev. Biol.* **197**, 248-69.

Vanderlaan, G., et al. (2005). Gli function is essential for motor neuron induction in zebrafish. *Dev. Biol.* **282**, 550-70.

Wang, H., et al. (2007). Isolation and expression of zebrafish zinc-finger transcription factor gene tsh1. *Gene Expr Patterns* **7**, 318-22.

Webb, K. J., et al. (2011). The Enhancer of split transcription factor Her8a is a novel dimerisation partner for Her3 that controls anterior hindbrain neurogenesis in zebrafish. *BMC Dev. Biol.* **11**, 27.

Woods, I. G., et al. (2005). The you gene encodes an EGF-CUB protein essential for Hedgehog signaling in zebrafish. *PLoS Biol.* **3**, e66.

Xu, Q., et al. (1995). Expression of truncated Sek-1 receptor tyrosine kinase disrupts the segmental restriction of gene expression in the Xenopus and zebrafish hindbrain. *Development* **121**, 4005-16.

Yao, J., et al. (2010). Atoh8, a bHLH transcription factor, is required for the development of retina and skeletal muscle in zebrafish. *PLoS One* **5**, e10945.

Yeo, S. Y., et al. (2001). Overexpression of a slit homologue impairs convergent extension of the mesoderm and causes cyclopia in embryonic zebrafish. *Dev. Biol.* **230**, 1-17.

Yu, H. H., et al. (2005). Semaphorin signaling guides cranial neural crest cell migration in zebrafish. *Dev. Biol.* **280**, 373-85.

Zannino, D. A., et al. (2014). prdm12b specifies the p1 progenitor domain and reveals a role for V1 interneurons in swim movements. *Dev. Biol.* **390**, 247-60.

Zhao, Q., et al. (2005). Expression of cyp26b1 during zebrafish early development. *Gene Expr Patterns* **5**, 363-9.

Zhu, S., et al. (2012). Activated ALK collaborates with MYCN in neuroblastoma pathogenesis. *Cancer Cell* **21**, 362-73.
