# Supplementary material for: A single cell transcriptome atlas of the developing zebrafish hindbrain
Source: Development. 2020 Mar 16;147(6):dev184143. doi: 10.1242/dev.184143 (PMC7097387; doi:10.1242/dev.184143)
Supplement: Reviewer comments [file develop-147-184143_review_history.pdf]

A single cell transcriptome atlas of the developing zebrafish hindbrain

Monica Tambalo, Richard Mitter and David G. Wilkinson
DOI: 10.1242/dev.184143

Editor: Stephen Wilson

Review timeline
Original submission: 23 August 2019
Editorial decision: 16 October 2019
First revision received: 17 December 2019
Editorial decision: 9 January 2020
Second revision received: 4 February 2020
Accepted: 11 February 2020

Original submission

First decision letter

MS ID#: DEVELOP/2019/184143

MS TITLE: A single cell transcriptome atlas of the developing zebrafish hindbrain
AUTHORS: Monica Tambalo, Richard Mitter, and David Wilkinson

Very sorry that it took so long to obtain reviews on your manuscript. The referees' comments are appended below, or you can access them online: please go to BenchPressand click on the 'Manuscripts with Decisions' queue in the Author Area.

As you will see, the referees express considerable interest in your work but have some constructive criticisms and suggestions for improving your manuscript. If you are able to revise the manuscript along the lines suggested, I will be happy receive a revised version of the manuscript. Please also note that Development will normally permit only one round of major revision.

Please attend to all of the reviewers' comments and ensure that you clearly highlight all changes made in the revised manuscript. Please avoid using 'Tracked changes' in Word files as these are lost in PDF conversion. I should be grateful if you would also provide a point-by-point response detailing how you have dealt with the points raised by the reviewers in the 'Response to Reviewers' box. If you do not agree with any of their criticisms or suggestions please explain clearly why this is so.

Reviewer 1

Advance summary and potential significance to field

In this manuscript, Tambalo et al. investigate the control of neurogenesis in the zebrafish hindbrain using single cell RNA sequencing at multiple stages of hindbrain development. Generating transcriptome datasets for three different stages, they then implement a variety of analytical approaches using this data to demonstrate its value as a resource.
4 main findings are presented:
i. Novel marker genes are identified for hindbrain segments, dorsovental cell types, and neuronal differentiation.

© 2020. Published by The Company of Biologists under the terms of the Creative Commons Attribution License (https://creativecommons.org/licenses/by/4.0/).

ii. Progressive changes in gene expression during the differentiation of precursor cells to neurons are found.
iii. Novel signaling factors are identified that are expressed in boundary cells or segment centres.
iv. The datasets are used to predict gene regulatory networks, with predicted interactions for scrt genes during neurogenesis being presented as an example.
The developing hindbrain and its adjacent tissues play fundamental roles during development of the vertebrate head, and serve as a model for investigating numerous embryological processes, including tissue patterning and differentiation. The transcriptome atlas of the developing zebrafish hindbrain provided by this work, will be an extremely valuable resource for other researchers, and the analyses presented in this manuscript highlight the utility of this resource. This work is well written, with the RNA-seq data analyses clearly presented and validated by high quality in-situ hybridization and sectioning data.

Comments for the author

This work fits well with the scope of Development and I only have one major suggestion for improvement.

Major points:
i. In the GRN analysis shown in Fig.7G, the authors produce a GRN for each individual stage using GENIE3, then focus on neurogenesis at 44hpf. This promises to be a powerful approach for guiding future functional interrogation of regulatory interactions. Since a fair amount is known about the GRN that governs rhombomeric gene expression at earlier stages, this seems to provide a good opportunity to compare the known GRN to the GRN generated by GENIE3 for the 16hpf data. This could provide an indication of the efficacy of the algorithm in capturing real gene regulatory interactions from the data, which would provide useful context for interpreting the predicted GRNs for the later stages.

<u>Reviewer 2</u>

Advance summary and potential significance to field

In this paper, Tambalo and colleagues carry out the most detailed single-cell transcriptomic analysis of the developing zebrafish hindbrain before, during, and after the patterning of neurogenesis. Their computational analysis reveals the expression of known marker genes in cells supposed to express them, and paves the way for (i) the study of genes not previously associated with different cell groups and (ii) at levels not previously studied before (i.e. whole cell transcriptome). Their meticulous work will be a useful resource for the developmental biology community and especially for those interested in hindbrain development and patterning of neurogenesis. The paper is certainly a good fit for Development!

Comments for the author

Comments
I suggest a number of small revisions to improve the paper and get it ready for publication in Development:

Related to the figures:
- In some of the figures (for example, in Fig. 6) the order of the panels is confusing and is difficult to find the panel the text is referring to. Also, not all figures appear in order in the text.
Maybe the authors can think of reorganising the panels/better labelling?
- In general, I find the labelling (especially gene names) to be too small – please use bigger font.
- Not all images are labelled with the stage. See for example, in Fig.2.
Including a "16 hpf"
label in the figure itself will make it easier for the reader – please revise this.
- All images are lacking scale bars – please include them.
- Some scales are missing (see for example Fig. 6U).
- Spelling out acronyms in the t-SNE plots will also make navigating them easier.

© 2020. Published by The Company of Biologists under the terms of the Creative Commons Attribution License (https://creativecommons.org/licenses/by/4.0/).

- Page 6. "The 16 hpf hindbrain is mainly constituted of progenitors (91% of total hindbrain cells; Fig.2A), and neurogenesis accounts for 6% of hindbrain cells." It seems that the authors mean that 6% of hindbrain cells are neurons/neurons at distinct differentiation states. If true please correct the text (and if appropriate correct also Fig. 2A).
- Fig. 2. It is interesting that the clustering performed splits r2-r4 and r7 in ventral and dorsal clusters, but not the other rhombomeres at 16 hpf. Could the authors comment on this? Because the clustering depends on user-defined parameters, how robust are the clusters shown? Maybe setting the clustering resolution a touch higher splits cells in r5 and r6 into dorsal and ventral clusters? It seems relevant to try this because the authors suggest that DV patterning is one of the main sources of transcriptome heterogeneity at this stage and indeed Fig. 2C shows that cells from all rhombomeres express genes associated with the dorsal domain (or at least zic2b).
- A number of times, the authors describe the position of clusters in their t-SNE plots to discuss the relationship between them (how similar/different clusters are based on their position in the t-SNE plot). For example, Page 10. "16 hpf and 24 hpf progenitors are in distinct clusters, but in close proximity in tSNE space, whereas 44 hpf progenitor cells are further apart (Fig.5C)". However, this global structure in t-SNE plots is known to be not meaningful because it can (and does) vary when run using different parameters. Please correct the text or use other means to study the relatedness of the different clusters. The authors may also want to try UMAP (see for example https://www.nature.com/articles/nbt.4314), which seems to preserve more of the global data structure than t-SNE and is gaining many adepts in the field!
- How many embryos were analysed for each in situ hybridisation experiment?
Please include this information in the figure legend.
- Page 9. "In contrast, there is no overlap of neurogenesis at 16 hpf and 24 hpf with differentiating cells at 44 hpf (C2 C7, C8), consistent with the generation of new neuronal sub-types.". What about the small clusters of cells from 24 hpf (in green in Fig. 5C) assigned to cluster 8 (Fig. 5A)?
- Page 11. "(...) while in C5 all cells are nonneurogenic.". I don't think the authors can talk about the neurogenic potential of these cells based on a snapshot of their transcriptomes. Better describe what they see "while in C5 all cells do not express pro-neural/neurogenic genes".
- Where will the data live and how can the community access it? I couldn't see any GEO accession number mentioned in the text, but I'm sure the authors are into it!

Minor comments
- Page 10. Gene ontology terms associated with the top 30 genes enriched in C0-C4 (16 hpf) and C3-C5 (24 hpf). This seems to suggest C0 to C4 and C3 to C5, better to write C0 and C4 and C3 and C5, or even better to write ventral and dorsal progenitors.
- Revise that the appropriate nomenclature for genes is used throughout the text.
- "Neural epithelium" is uncommon, why not use the more broadly used term "neuroepithelium"?
- Dorsoventral and D-V are randomly used throughout the text, but the authors could simply write D-V after introducing the well-known acronym.
- Arrows in figure images pointing to the population of interest will be helpful.
- Page 11 In addition, we find new genes with expression enriched at boundaries including rnd2, dystroglycan 1 (Parsons et al., 2002) and the BMP inhibitor follistatin 1b (Fig.2F; Dal-Pra et al., 2006). A small typo, this should refer to Fig. 6F.
- Page 20. "Lowly expressed genes with a count of >=1" . I guess the authors mean count of <=1?

<u>Reviewer 3</u>

Advance summary and potential significance to field

Tambalo et al. report a transcriptomic atlas of the developing zebrafish hindbrain using single cell RNA-seq at 16, 24 and 48 hours of development. The paper will provide a useful resource after the following points are addressed.

Comments for the author

1. There are differing numbers of clusters at different stages. Is this the result of over/under-sequencing or over/under-clustering or is there a biological meaningful change in the molecular composition of the hindbrain between these different time points that results in gain or loss of cell types? What uniform criteria were used to define the optimum number of clusters in these stages?

© 2020. Published by The Company of Biologists under the terms of the Creative Commons Attribution License (https://creativecommons.org/licenses/by/4.0/).

2. For the neuronal clusters, the authors should include some analysis on how the clusters from different stages might map to one another.

3. '…16 hpf and 24 hpf progenitors are in distinct clusters, but in close proximity in tSNE space, whereas 44 hpf progenitor cells are further apart…'
– tSNE is a dimensionality reduction technique that is well known to produce distortions in Euclidean space. Therefore, the distance between clusters or cluster size often means little in t-SNE space (UMAP is better suited for this kind of analysis). The authors should not make claims about proximity of clusters based on proximity of cluster dots in the t-SNE space. A detailed dendrogram in the variable genes space should be presented to reveal proximity of clusters.

4. Since the authors are aggregating the three time points, it should be clarified whether these timepoints were collected in the same sequencing batch. Otherwise, it is unclear how to deconvolute biological differences from technical variability.

5. It is interesting that there are three classes of progenitors. Do these give rise to distinct mature neuronal populations?

6. '…We bioinformatically isolated 24 hpf ventral progenitors and used rfng (boundary), etv5b (segment center), and neurog1 and neurod4 (neuronal differentiation) to drive clustering. Seven sub-clusters were obtained..' – In this supervised approach, did the authors only use these four genes to cluster these cells? Perhaps the clustering should be done on a larger gene set that distinguishes these different types of cells along the boundary segments.

7. The authors mention that "…At 16 hpf, clustering is driven by segmental and dorsoventral identity (Fig.2A), whereas at 24 hpf and 44 hpf cells are clustered by dorsoventral identity and differentiation state…" citing the tSNE plot on Figure 2. However, the segmental and dorsoventral identity in Figure 2A do not seem to be driving the entirety of the clustering results. It seems that cells in the same rhombomere might belong to two clusters.

8. Figure 2C: It is unclear why those particular genes are shown in the plots. The authors cite these as cluster specific markers in the text but their expression is clearly distributed across multiple clusters. In general, the authors should nominate specific markers for all of the clusters presented at different stages. If there are no single genes that define each cluster, a combinatorial expression of multiple genes should be used to mark these clusters.

9. Figure 2D: The genes listed in Panel D and E should be shown in context of the clusters from single cell data, either in the form of a dot plot, violin plot or feature plots as in C.

10. All of the feature plots need labels for expression level.

11. All feature plots should also be labeled with the cluster identities that were intended to be highlighted by the authors.

12. The paper would benefit from the addition of a schematic of the hindbrain at the 3 stages that were used for profiling, along with the expression domains of the different clusters obtained at those stages.

13. The paper would also benefit from clear naming conventions that follows a theme throughout the paper. Currently, there are numbers representing different clusters in different figures but they don't seem to follow a common thread.

14. The in situ hybridization panels in the figures are not annotated with cluster id or contextual information on why the panel is presented. Similarly, many of the situs presented in the paper do not seem to be spatially restricted. A zoomed in picture with clear demarcation of expression should be provided.

© 2020. Published by The Company of Biologists under the terms of the Creative Commons Attribution License (https://creativecommons.org/licenses/by/4.0/).

First revision

Author response to reviewers' comments

We thank the reviewers for their constructive comments. We have addressed the points by carrying out new analyses, and modifications to the text and figure, as detailed below. These changes have significantly improved the manuscript.

Reviewer 1 Advance summary and potential significance to field
In this manuscript, Tambalo et al. investigate the control of neurogenesis in the zebrafish hindbrain using single cell RNA sequencing at multiple stages of hindbrain development. Generating transcriptome datasets for three different stages, they then implement a variety of analytical approaches using this data to demonstrate its value as a resource.
4 main findings are presented:
i. Novel marker genes are identified for hindbrain segments, dorsovental cell types, and neuronal differentiation.
ii. Progressive changes in gene expression during the differentiation of precursor cells to neurons are found.
iii. Novel signaling factors are identified that are expressed in boundary cells or segment centres.
iv. The datasets are used to predict gene regulatory networks, with predicted interactions for scrt genes during neurogenesis being presented as an example.

The developing hindbrain and its adjacent tissues play fundamental roles during development of the vertebrate head, and serve as a model for investigating numerous embryological processes, including tissue patterning and differentiation. The transcriptome atlas of the developing zebrafish hindbrain, provided by this work, will be an extremely valuable resource for other researchers, and the analyses presented in this manuscript highlight the utility of this resource. This work is well written, with the RNA-seq data analyses clearly presented and validated by high quality in-situ hybridization and sectioning data.

Reviewer 1 Comments for the author
This work fits well with the scope of Development and I only have one major suggestion for improvement.

Major points:
i. In the GRN analysis shown in Fig.7G, the authors produce a GRN for each individual stage using GENIE3, then focus on neurogenesis at 44hpf. This promises to be a powerful approach for guiding future functional interrogation of regulatory interactions. Since a fair amount is known about the GRN that governs rhombomeric gene expression at earlier stages, this seems to provide a good opportunity to compare the known GRN to the GRN generated by GENIE3 for the 16hpf data. This could provide an indication of the efficacy of the algorithm in capturing real gene regulatory interactions from the data, which would provide useful context for interpreting the predicted GRNs for the later stages.

This is a good idea and we now present a GENIE3 analysis of the 16 hpf data (new Suppl. Fig. 7, presented on p.15). GENIE3 predicts interactions between egr2b, mafba, epha4 and multiple hox genes, including seven interactions which have previously been validated in vivo.

Reviewer 2 Advance summary and potential significance to field
In this paper, Tambalo and colleagues carry out the most detailed single-cell transcriptomic analysis of the developing zebrafish hindbrain before, during, and after the patterning of neurogenesis. Their computational analysis reveals the expression of known marker genes in cells supposed to express them, and paves the way for (i) the study of genes not previously associated with different cell groups and (ii) at levels not previously studied before (i.e. whole cell transcriptome). Their meticulous work will be a useful resource for the developmental biology community and especially for those interested in hindbrain development and patterning of neurogenesis. The paper is certainly a good fit for Development!

© 2020. Published by The Company of Biologists under the terms of the Creative Commons Attribution License (https://creativecommons.org/licenses/by/4.0/).

Reviewer 2 Comments for the author
Comments
I suggest a number of small revisions to improve the paper and get it ready for publication in Development:

Related to the figures:
- In some of the figures (for example, in Fig. 6) the order of the panels is confusing and is difficult to find the panel the text is referring to. Also, not all figures appear in order in the text. Maybe the authors can think of reorganising the panels/better labelling?

We have reorganised the panels to bring together those that present related data. The overall organisation is presented in the first part of the relevant results, and we have now improved the links between the text and individual panels. We have also added more annotation, indicated features of interest with arrowheads, and used a colour code to indicate the different representations of the same cluster. We organised Fig. 6 so that related data are adjacent. For example, projection plots of boundary genes in (C), and in situ hybridisations in (D-F); projection plots of segment centre genes in (G) and in situ hybridisations in (H-P). The 44 hpf UMAP, projection plots and heat maps are in (R-U).

- In general, I find the labelling (especially gene names) to be too small – please use bigger font.

The font size is small in the dot plots in which many genes are represented. We have reorganised the figures so that these dot plots occupy a full page width and the font size is increased.

- Not all images are labelled with the stage. See for example, in Fig.2. Including a "16 hpf" label in the figure itself will make it easier for the reader – please revise this.

We have added the stage to the relevant Figures, together with an illustration of the main features (segments, D-V domains) that are driving the clustering.

- All images are lacking scale bars – please include them.

We have added scale bars.

- Some scales are missing (see for example Fig. 6U).

We have added scales.

- Spelling out acronyms in the t-SNE plots will also make navigating them easier.

We have avoided acronyms where possible, but in some figures it is difficult to accommodate the full names. We have given a key to the acronyms on the figure or in the legend.

- Page 6. "The 16 hpf hindbrain is mainly constituted of progenitors (91% of total hindbrain cells; Fig.2A), and neurogenesis accounts for 6% of hindbrain cells." It seems that the authors mean that 6% of hindbrain cells are neurons/neurons at distinct differentiation states. If true, please correct the text (and if appropriate correct also Fig. 2A).

We have changed the text on p.6 to make clear that we are referring to cells at different stages of neurogenesis (neuroD4 and later marker expression).

- Fig. 2. It is interesting that the clustering performed splits r2-r4 and r7 in ventral and dorsal clusters, but not the other rhombomeres at 16 hpf. Could the authors comment on this?

Because the clustering depends on user-defined parameters, how robust are the clusters shown? Maybe setting the clustering resolution a touch higher splits cells in r5 and r6 into dorsal and ventral clusters? It seems relevant to try this because the authors suggest that DV patterning is one of the main sources of transcriptome heterogeneity at this stage and indeed Fig. 2C shows that cells from all rhombomeres express genes associated with the dorsal domain (or at least zic2b).

© 2020. Published by The Company of Biologists under the terms of the Creative Commons Attribution License (https://creativecommons.org/licenses/by/4.0/).

This is an interesting point. Markers such as zic2b demarcate the D-V differences, but not all rhombomeres are split into dorsal and ventral clusters by Seurat. Presumably this reflects quantitative difference in the transcriptome within distinct segments. As suggested, we have changed the clustering resolution and find that this splits all rhombomeres into dorsal and ventral domains. Interestingly, this also generates further clusters. We present this Fig. S4 and discuss on p.7.

- A number of times, the authors describe the position of clusters in their t-SNE plots to discuss the relationship between them (how similar/different clusters are based on their position in the t-SNE plot). For example, Page 10. "16 hpf and 24 hpf progenitors are in distinct clusters, but in close proximity in tSNE space, whereas 44 hpf progenitor cells are further apart (Fig.5C)". However, this global structure in t-SNE plots is known to be not meaningful because it can (and does) vary when run using different parameters. Please correct the text or use other means to study the relatedness of the different clusters. The authors may also want to try UMAP (see for example https://www.nature.com/articles/nbt.4314), which seems to preserve more of the global data structure than t-SNE and is gaining many adepts in the field!

Thank you for this helpful idea. We have changed all of the plots from t-SNE to UMAP. We find that many of the relationships are similar, such as the relative ordering of clusters with distinct segmental identity. However, UMAP presents a different picture of some of the other relationships. We have extensively revised the text accordingly.

- How many embryos were analysed for each in situ hybridisation experiment?

At least 30 embryos were analysed for each gene, and in situ hybridisations were carried out in two separate experiments. We now mention this in the Methods.

Please include this information in the figure legend.
- Page 9. "In contrast, there is no overlap of neurogenesis at 16 hpf and 24 hpf with differentiating cells at 44 hpf (C2, C7, C8), consistent with the generation of new neuronal sub-types.". What about the small clusters of cells from 24 hpf (in green in Fig. 5C) assigned to cluster 8 (Fig. 5A)?

This is a good point, which also applies to the new analysis using UMAP. There is some overlap which suggests that some neurons present at 44 hpf have started to be generated at the earlier stages. We have changed the text on p.9 to take account of this.

- Page 11. "(...) while in C5 all cells are nonneurogenic.". I don't think the authors can talk about the neurogenic potential of these cells based on a snapshot of their transcriptomes. Better describe what they see "while in C5 all cells do not express pro-neural/neurogenic genes".

This is a good point – we had defined neurogenic versus non-neurogenic zones based on expression of proneural genes required for neurogenesis, but have no data to show their differentiation potential. We have clarified this definition on p.11 and changed the text as suggested.

- Where will the data live and how can the community access it? I couldn't see any GEO accession number mentioned in the text, but I'm sure the authors are into it!

Our data are now deposited in GEO GSE141428 and are available at the Single Cell Portal https://singlecell.broadinstitute.org/single_cell/study/SCP667/a-single-cell-transcriptome-atlas-of-the-developing-zebrafish-hindbrain#study-summary.
The R analysis script developed for this paper is available at https://github.com/crickbabs/ZebrafishDevelopingHindbrainAtlas.

Minor comments
- Page 10. Gene ontology terms associated with the top 30 genes enriched in C0-C4 (16 hpf) and C3-C5 (24 hpf). This seems to suggest C0 to C4 and C3 to C5, better to write C0 and C4 and C3 and C5, or even better to write ventral and dorsal progenitors.

We have made this change.

© 2020. Published by The Company of Biologists under the terms of the Creative Commons Attribution License (https://creativecommons.org/licenses/by/4.0/).

- Revise that the appropriate nomenclature for genes is used throughout the text.

We have made changes to conform to the appropriate gene nomenclature.

- "Neural epithelium" is uncommon, why not use the more broadly used term "neuroepithelium"?

We have made this change.

- Dorsoventral and D-V are randomly used throughout the text, but the authors could simply write D-V after introducing the well-known acronym.

We have made this change.

- Arrows in figure images pointing to the population of interest will be helpful.

We have added arrows as suggested.

- Page 11 In addition, we find new genes with expression enriched at boundaries including rnd2, dystroglycan 1 (Parsons et al., 2002) and the BMP inhibitor follistatin 1b (Fig.2F; Dal-Pra et al., 2006). A small typo, this should refer to Fig. 6F.

We have corrected this.

- Page 20. "Lowly expressed genes with a count of >=1" . I guess the authors mean count of <=1?

We have corrected this.

Reviewer 3 Advance summary and potential significance to field
Tambalo et al. report a transcriptomic atlas of the developing zebrafish hindbrain using single cell RNA-seq at 16, 24 and 48 hours of development. The paper will provide a useful resource after the following points are addressed.

Reviewer 3 Comments for the author
There are differing numbers of clusters at different stages. Is this the result of over/under-sequencing or over/under-clustering or is there a biological meaningful change in the molecular composition of the hindbrain between these different time points that results in gain or loss of cell types? What uniform criteria were used to define the optimum number of clusters in these stages?

We were guided by previous knowledge of the biology to optimise the number of clusters, so that they give a useful representation of the data. There is a major increase in the number of neurons and complexity of neuronal cell types during the period under study, and this underlies the increasing number of clusters. At 16 hpf, positional information has been defined along the A-P and D-V axes of the progenitor cells in the hindbrain, and neurogenesis to form the early-born neuronal cell types has only just started (6% of hindbrain cells). Later-born cell types are generated during the next several days, and consequently there is an increasingly complex set of neurons, which by 44 hpf comprise 55% of hindbrain cells. The optimum number of clusters depends on the specific features of interest, and will depend on how many transcriptomic differences are used to define cells as distinct from each other. For example, in Fig.2, r5 cells formed a distinct cluster, whereas r2, r3 and r4 cells were in the same cluster but can be distinguished by specific markers, such as egr2. Likewise, cells with distinct D-V identity were only partly segregated into different clusters. We have tested the effect of adjusting the criteria, and can split all rhombomeres into dorsal and ventral populations, and r3 from r2+r4; this is presented in new Fig. S4. Our aim in this study is to present the overall picture revealed by major changes in transcriptome at the three stages analysed. The data are a resource for further analysis of subsets of cells to obtain a finer-grained separation, and this is likely to reveal many more cell states.

2. For the neuronal clusters, the authors should include some analysis on how the clusters from different stages might map to one another.

© 2020. Published by The Company of Biologists under the terms of the Creative Commons Attribution License (https://creativecommons.org/licenses/by/4.0/).

This is an interesting question, which we tried to address in the analysis of aggregated data from the three stages (Fig.5). There is only a small overlap between the differentiated cells at the three stages, which reflects that distinct neuronal cell types are sequentially generated. A more refined analysis at closer-spaced time intervals would be needed to trace the differentiation of the specific cell types that are detected at 44 hpf.

3. '…16 hpf and 24 hpf progenitors are in distinct clusters, but in close proximity in tSNE space, whereas 44 hpf progenitor cells are further apart…'
- tSNE is a dimensionality reduction technique that is well known to produce distortions in Euclidean space. Therefore, the distance between clusters or cluster size often means little in t-SNE space (UMAP is better suited for this kind of analysis). The authors should not make claims about proximity of clusters based on proximity of cluster dots in the t-SNE space. A detailed dendrogram in the variable genes space should be presented to reveal proximity of clusters.

We have changed all of the projections to UMAP as this better represents the relationship between the clusters. We find that many of the relationships are similar, such as the relative ordering of clusters with distinct segmental identity. However, UMAP presents a different picture of some of the other relationships. We have extensively revised the text accordingly.

4. Since the authors are aggregating the three time points, it should be clarified whether these timepoints were collected in the same sequencing batch. Otherwise, it is unclear how to deconvolute biological differences from technical variability.

The time points were not collected in the same sequencing batch. Our in situ hybridisation studies, as well as the extensive literature on sequential gene expression during neuronal differentiation, give a robust biological validation of the findings. For example, cell type specific markers of late-born neurons present at 44 hpf are not detected at 16 hpf; there is a shift in the proportion of cells expressing proneural (e.g. neurog1, neuroD4) and downstream genes (elavl4) that reflects the cascade of neuronal maturation; mir9 genes are expressed in progenitors at 44 hpf but not at the earlier stages; proliferation markers that are widely expressed at early stages are spatially restricted by 44 hpf.

5. It is interesting that there are three classes of progenitors. Do these give rise to distinct mature neuronal populations?

This is an interesting question as it is currently not known whether they give rise to distinct neurons. We have clarified this on page 4. The progenitors are defined here based on gene expression, and lineage analyses to determine the neuronal types they generate have not been carried out. Proneural genes which drive neurogenesis are widely expressed at 16 hpf, and after 24 hpf become confined to zones flanking the hindbrain boundaries. It is likely that these generate the great majority of neuronal cell types formed during the period under study. Neurogenesis is inhibited at boundaries by Notch and Yap/Taz activation, but recent work has shown that after 40 hpf these start to generate some neurons. Neurogenesis is inhibited at segment centres by Fgf20 signaling. The current view is that this switches the progenitors to gliogenesis, but further work will be required to verify this and identify the cell types that are generated.

6. '…We bioinformatically isolated 24 hpf ventral progenitors and used rfng (boundary), etv5b (segment center), and neurog1 and neurod4 (neuronal differentiation) to drive clustering. Seven sub-clusters were obtained..' – In this supervised approach, did the authors only use these four genes to cluster these cells? Perhaps the clustering should be done on a larger gene set that distinguishes these different types of cells along the boundary segments.

We confirm that we used these four genes to drive the clustering. The purpose of the analysis is that there are very few genes known to be specifically expressed in boundary cells or segment centres, and we wanted to use the scRNA data to find further markers. In previous work we showed that Fgf20 signaling upregulates the genes that are specifically expressed in segment centres. We chose etv5b as it is a direct target of Fgf signaling. With regard to boundary cells, rfng is one of very few genes known to be specifically expressed in these cells (the other is an adjacent gene that is co-regulated). The use of these genes to drive the clustering succeeded in finding novel markers for the boundary cell and segment centre progenitors.

© 2020. Published by The Company of Biologists under the terms of the Creative Commons Attribution License (https://creativecommons.org/licenses/by/4.0/).

7. The authors mention that "…At 16 hpf, clustering is driven by segmental and dorsoventral identity (Fig.2A), whereas at 24 hpf and 44 hpf cells are clustered by dorsoventral identity and differentiation state…" citing the tSNE plot on Figure 2. However, the segmental and dorsoventral identity in Figure 2A do not seem to be driving the entirety of the clustering results. It seems that cells in the same rhombomere might belong to two clusters.

Our interpretation of the results is that the clustering is driven by segmental and dorsoventral identity, apart from the one cluster corresponding to neurogenesis. Cells in the same rhombomere can be subdivided into two clusters that have distinct dorsoventral identity.

8. Figure 2C: It is unclear why those particular genes are shown in the plots. The authors cite these as cluster specific markers in the text but their expression is clearly distributed across multiple clusters. In general, the authors should nominate specific markers for all of the clusters presented at different stages. If there are no single genes that define each cluster, a combinatorial expression of multiple genes should be used to mark these clusters.

We have improved the description of these data. The genes were chosen because they are well characterised markers (and regulators) of distinct cell states along the D-V (zic2, nkx2.2b) and A-P axes (hoxa2b, egr2b, mafba, hoxd4a, eng2a) and of the cascade of neuronal differentiation (neurog1, neurod4). As the reviewer points out, most markers do not define a single cluster. This reflects that identity is regulated by a combination of genes. We list genes expressed in different segments and D-V domains in D and E, which include known markers that enable the identity of all clusters to be assigned. For example, along the A-P axis, hoxd4 is expressed in r7, mafb in r6 and r5, egr2b in r5 and r3, hoxa2b in r2-r5, eng2a in MHB-r1.

9. Figure 2D: The genes listed in Panel D and E should be shown in context of the clusters from single cell data, either in the form of a dot plot, violin plot or feature plots as in C.

The genes listed in D and E give a more comprehensive description of the differences between the clusters. Many of these are well characterised markers that are known to be co-expressed, for example ephA4 and egr2 in r3 and r5; eng2a, eng2b, fgf8a, pax2a in r1. We chose to display representative markers rather than all of them on feature plots, as otherwise the figure would be very large and duplicate published information. Heat maps of these genes are presented in Fig. S3.

10. All of the feature plots need labels for expression level.

We have added labels for expression levels.

11. All feature plots should also be labeled with the cluster identities that were intended to be highlighted by the authors.

We have introduced a colour coding to link the clusters in part A to the other figure parts. In B, a dot with same colour is adjacent to the cluster number, and in C coloured arrowheads indicate the clusters in which the indicated gene has high level expression.

12. The paper would benefit from the addition of a schematic of the hindbrain at the 3 stages that were used for profiling, along with the expression domains of the different clusters obtained at those stages.

We have added a schematic of the expression domains to the figures.

13. The paper would also benefit from clear naming conventions that follows a theme throughout the paper. Currently, there are numbers representing different clusters in different figures but they don't seem to follow a common thread.

The numbers are assigned by the analysis program in ranking order of the number of cells in the cluster, and we are using them as a way to refer to the cluster independently of its identity. As the number and nature of the clusters varies between time points, they cannot be linked directly. For example, segmental identity drives clustering at 16 hpf but not 24 hpf or 44 hpf. In principle, links

© 2020. Published by The Company of Biologists under the terms of the Creative Commons Attribution License (https://creativecommons.org/licenses/by/4.0/).

could be made for clusters along the dorsoventral axis, but this would require a closer spaced time series. We agree that it would be good to have a common nomenclature to describe the clusters. We are using the terms usually used to describe the different populations: progenitors, dorsal, ventral, roof plate, floor plate, neurogenesis, and at 44 hpf the names of specific neuronal cell types that have formed by that stage.

14. The in situ hybridization panels in the figures are not annotated with cluster id or contextual information on why the panel is presented. Similarly, many of the situs presented in the paper do not seem to be spatially restricted. A zoomed in picture with clear demarcation of expression should be provided.

All of the in situs show genes that are spatially restricted, except proliferation markers that are widely expressed at early stages. We now label the relevant features in the in situs, and use a colour code for the arrow heads to link them to the feature plots and UMAP clusters. We have now better explained why the gene expression patterns are presented.

Second decision letter

MS ID#: DEVELOP/2019/184143

MS TITLE: A single cell transcriptome atlas of the developing zebrafish hindbrain

AUTHORS: Monica Tambalo, Richard Mitter, and David G. Wilkinson

Happy new year. The reviewers are happy with your revisions and there are just a couple of very minor points to consider before publication. The referees' comments are appended below, or you can access them online: please go to BenchPress and click on the 'Manuscripts with Decisions' queue in the Author Area. Please attend to all of the reviewers' comments in your revised manuscript.

Reviewer 1

Advance summary and potential significance to field

As stated in my review of the original submission: The transcriptome atlas of the developing zebrafish hindbrain, provided by this work, will be an extremely valuable resource for other researchers, and the analyses presented in this manuscript highlight the utility of this resource. This work is well written with the RNA-seq data analyses clearly presented and validated by high quality in-situ hybridization and sectioning data. This work fits well with the scope of Development

Comments for the author

The revised manuscript provides an enhanced set of analyses and considerable modifications to the text and figures in response to the specific points raised by all of the reviewers. They have included an informative analysis comparing their data with existing models for hindbrain GRNs, as I requested. I also feel that the changes in response to other reviewers points has really strengthen the study and added more clarity to their findings and interpretations. I feel this is high quality work that will be a useful resource for the community and support publication as it stands.

Reviewer 2

Advance summary and potential significance to field

In this paper, Tambalo and colleagues carry out the most detailed single-cell transcriptomic analysis of the developing zebrafish hindbrain before, during, and after the patterning of

© 2020. Published by The Company of Biologists under the terms of the Creative Commons Attribution License (https://creativecommons.org/licenses/by/4.0/).

neurogenesis. Their computational analysis reveals the expression of known marker genes in the cells supposed to express them, and paves the way for (i) the study of genes not previously associated with different cell groups and (ii) at levels not previously studied before (i.e. whole cell transcriptome). Their meticulous work will be a useful resource for the developmental biology community and especially for those interested in hindbrain development and patterning of neurogenesis.

Comments for the author

Tambalo and colleagues have much improved this revised version of their manuscript. The figures are now more complete and clearer, and their description of the data/computational analysis is more precise. I have two minor comments and a suggestion for presenting their clustering analysis/conclusions but, overall, I would like to read this paper in Development!
- A small detail that will be good to correct. Page 5 says "In total, 9026 cells were sequenced (...). After applying the appropriate Seurat quality filters, a total of 2929, 2568 and 3529 cells (...) were retained for analysis". But based on this the total number of cells after filtering equals the total number of cells sequenced, and this should be higher (the number of cells that passed QC plus the number of cells that didn't).
- Page 15: " In the next group, a subset of genes initiates expression that then declines late in pseudotime (G5)." Doesn't the expression increase late in pseudotime?
- As described in page 7, increasing the clustering resolution (Fig S4) seems to split the cells into D-V identities and much better into rhombomere identity than the clustering resolution chosen for Fig 2. Why not showing the UMAP plot with the higher clustering resolution in the main figure and keeping the text simpler? I think that if the authors want to discuss/show cluster similarities, they will be better plotting clusters as a tree (check the PlotClusterTree function in Seurat) rather than supporting their conclusions with a few genes and "UMAP distance" (even if more informative than "t-SNE distance").

Reviewer 3

Advance summary and potential significance to field

The authors have addressed my suggestions.

Comments for the author

The authors have addressed my suggestions.

Second revision

Author response to reviewers' comments

We thank the reviewers for their further comments on our manuscript. We have addressed the points raised by Reviewer 2 as summarised below.

Reviewer 1 Advance summary and potential significance to field
As stated in my review of the original submission: The transcriptome atlas of the developing zebrafish hindbrain, provided by this work, will be an extremely valuable resource for other researchers, and the analyses presented in this manuscript highlight the utility of this resource. This work is well written, with the RNA-seq data analyses clearly presented and validated by high quality in-situ hybridization and sectioning data. This work fits well with the scope of Development

Reviewer 1 Comments for the author
The revised manuscript provides an enhanced set of analyses and considerable modifications to the text and figures in response to the specific points raised by all of the reviewers. They have included an informative analysis comparing their data with existing models for hindbrain GRNs, as I

© 2020. Published by The Company of Biologists under the terms of the Creative Commons Attribution License (https://creativecommons.org/licenses/by/4.0/).

requested. I also feel that the changes in response to other reviewers points has really strengthen the study and added more clarity to their findings and interpretations. I feel this is high quality work that will be a useful resource for the community and support publication as it stands.

Reviewer 2 Advance summary and potential significance to field

In this paper, Tambalo and colleagues carry out the most detailed single-cell transcriptomic analysis of the developing zebrafish hindbrain before, during, and after the patterning of neurogenesis. Their computational analysis reveals the expression of known marker genes in the cells supposed to express them, and paves the way for (i) the study of genes not previously associated with different cell groups and (ii) at levels not previously studied before (i.e. whole cell transcriptome). Their meticulous work will be a useful resource for the developmental biology community and especially for those interested in hindbrain development and patterning of neurogenesis.

Reviewer 2 Comments for the author

Tambalo and colleagues have much improved this revised version of their manuscript. The figures are now more complete and clearer, and their description of the data/computational analysis is more precise. I have two minor comments and a suggestion for presenting their clustering analysis/conclusions but, overall, I would like to read this paper in Development!

- A small detail that will be good to correct. Page 5 says "In total, 9026 cells were sequenced (...). After applying the appropriate Seurat quality filters, a total of 2929, 2568 and 3529 cells (...) were retained for analysis". But based on this the total number of cells after filtering equals the total number of cells sequenced, and this should be higher (the number of cells that passed QC plus the number of cells that didn't).

We have changed the text on page 5 to correct this. In the new UMAP analysis, instead of eliminating cells based on mitochondrial content, total counts or total expressed genes, all cells were used without filtering. Cells that were outliers for these metrics were flagged and found not to behave oddly or drive any clustering. We therefore did not apply the Seurat filter and there was no change in cell number.

- Page 15: " In the next group, a subset of genes initiates expression that then declines late in pseudotime (G5)." Doesn't the expression increase late in pseudotime?

Thank you, we have corrected this error.

- As described in page 7, increasing the clustering resolution (Fig S4) seems to split the cells into D-V identities and much better into rhombomere identity than the clustering resolution chosen for Fig 2. Why not showing the UMAP plot with the higher clustering resolution in the main figure and keeping the text simpler? I think that if the authors want to discuss/show cluster similarities, they will be better plotting clusters as a tree (check the PlotClusterTree function in Seurat) rather than supporting their conclusions with a few genes and "UMAP distance" (even if more informative than "t-SNE distance").

We have as suggested now carried out and present PlotClusterTree analysis of the 16 hpf data. This is presented in Fig.S4 and discussed on page 8. The findings largely fit with the inferences from the UMAP plots. However, as D-V identity dominates in the segregation of cells in r2+r3+r4, the tree does not reveal the similarities in r3 and r5 gene expression suggested by previous work and suggested by the two-dimensional UMAP plot. We have kept the higher resolution UMAP plus PlotClusterTree analysis as Supplemental Figure 4 for two reasons:

It is difficult to add the PlotClusterTree to main Fig.2 as it is already large and complex, and we feel the existing figure parts all need to be retained.

In addition to increasing the segregation of cells by D-V identity, the higher resolution analysis creates further subdivisions based on cell cycle genes. As expression of these genes is not spatially localised at this stage, this likely represents heterogeneous expression within the neuroepithelial cells, though it is unclear why this further clustering is restricted to r2+r4 cells. We prefer to

© 2020. Published by The Company of Biologists under the terms of the Creative Commons Attribution License (https://creativecommons.org/licenses/by/4.0/).

present the simpler lower resolution picture of segregation based on segmental, D-V and differentiation in the main figure.

Reviewer 3 Advance summary and potential significance to field
The authors have addressed my suggestions.

Reviewer 3 Comments for the author
The authors have addressed my suggestions.

Third decision letter

MS ID#: DEVELOP/2019/184143

MS TITLE: A single cell transcriptome atlas of the developing zebrafish hindbrain

AUTHORS: Monica Tambalo, Richard Mitter, and David G. Wilkinson
ARTICLE TYPE: Techniques and Resources Article

I am happy to tell you that your manuscript has been accepted for publication in Development, pending our standard ethics checks.

© 2020. Published by The Company of Biologists under the terms of the Creative Commons Attribution License (https://creativecommons.org/licenses/by/4.0/).
